# Cholangiocarcinoma combined with biliary obstruction: an exosomal circRNA signature for diagnosis and early recurrence monitoring

**DOI:** 10.1038/s41392-024-01814-3

**Published:** 2024-05-03

**Authors:** Ningyuan Wen, Dingzhong Peng, Xianze Xiong, Geng Liu, Guilin Nie, Yaoqun Wang, Jianrong Xu, Shaofeng Wang, Sishu Yang, Yuan Tian, Bei Li, Jiong Lu, Nansheng Cheng

**Affiliations:** 1 Division of Biliary Surgery, Department of General Surgery, West China Hospital, Sichuan University, Chengdu, Sichuan China; 2 Research Center for Biliary Diseases, West China Hospital, Sichuan University, Chengdu, Sichuan China

**Keywords:** Prognostic markers, Tumour biomarkers

## Abstract

Cholangiocarcinoma (CCA) is a highly malignant biliary tract cancer with currently suboptimal diagnostic and prognostic approaches. We present a novel system to monitor CCA using exosomal circular RNA (circRNA) via serum and biliary liquid biopsies. A pilot cohort consisting of patients with CCA-induced biliary obstruction (CCA-BO, *n* = 5) and benign biliary obstruction (BBO, *n* = 5) was used to identify CCA-derived exosomal circRNAs through microarray analysis. This was followed by a discovery cohort (*n* = 20) to further reveal a CCA-specific circRNA complex (hsa-circ-0000367, hsa-circ-0021647, and hsa-circ-0000288) in both bile and serum exosomes. In vitro and in vivo studies revealed the three circRNAs as promoters of CCA invasiveness. Diagnostic and prognostic models were established and verified by two independent cohorts (training cohort, *n* = 184; validation cohort, *n* = 105). An interpreter-free diagnostic model disclosed the diagnostic power of biliary exosomal circRNA signature (Bile-DS, AUROC = 0.947, RR = 6.05) and serum exosomal circRNA signature (Serum-DS, AUROC = 0.861, RR = 4.04) compared with conventional CA19-9 (AUROC = 0.759, RR = 2.08). A prognostic model of CCA undergoing curative-intent surgery was established by calculating early recurrence score, verified with bile samples (Bile-ERS, C-index=0.783) and serum samples (Serum-ERS, C-index = 0.782). These models, combined with other prognostic factors revealed by COX-PH model, enabled the establishment of nomograms for recurrence monitoring of CCA. Our study demonstrates that the exosomal triple-circRNA panel identified in both bile and serum samples serves as a novel diagnostic and prognostic tool for the clinical management of CCA.

## Introduction

Cholangiocarcinoma (CCA) is a highly invasive malignancy with its incidence and mortality rising worldwide, and is classified into a diverse group of subtypes according to anatomical origins, namely intrahepatic CCA (iCCA), perihilar CCA (pCCA) and distal CCA (dCCA).^1,2^ The onset of CCA can be occult, under most conditions when patients present with painless progressive jaundice, they are already in the advanced stage and miss the opportunity for early intervention.^3^ Radical surgery is currently the only definite treatment for CCA, but its efficacy is attenuated due to the high incidence of early recurrence.^4^ To date, accurate preoperative assessment of CCA mainly relies on imaging techniques, with relatively low diagnostic power particularly for small invasive lesions; and tissue biopsy, which remains controversial particularly in surgical resection candidates.^5–7^ Meanwhile, carbohydrate antigen 19-9 (CA19-9), as the most commonly used biomarker for CCA diagnosis, has limited diagnostic power due to its high false-positive rate especially in patients with obstructive jaundice.^8^ Besides, there is a lack of prognostic tools for CCA. Particularly, as CCA tends to have early recurrence following curative-intent resection, there is a demand to identify patients who are most likely to benefit from surgical intervention. Therefore, it is vital to expand the diagnostic and prognostic toolbox to improve the clinical management of CCA.

Exosomes are extracellular nanoparticles with a diameter of 40–160 nm, and were found to be closely engaged with the progression of cancers.^9^ Enclosed within a lipid bilayer structure, exosomal cargos are highly anticipated biomarkers to reflect the state of their tissue of origin, especially when there is malignant transformation. Various kinds of exosomal contents were extracted and analyzed, whereas in the noncoding RNA (ncRNA) families, a special type of endogenous ring-structured ncRNA named circular RNA (circRNA) has drawn researchers’ attention. Owing to their structural stability, evolutionary conservation and significant enrichment in extracellular vesicles, exosomal circRNAs were considered to be ideal biomarkers in the diagnosis and prognosis prediction of malignancies.^10^ In recent studies, researchers have identified specific exosomal circRNAs closely associated with the CCA microenvironment, such as circ-CCAC1, circ-PTPN22, and circ-ADAMTS6.^11,12^ Functionally, exosomes derived from CCA cells and CCA-related microenvironment were found to be enriched with oncogenic cargos including the above-mentioned circRNAs, facilitating intercellular communication within the tumor microenvironment and driving tumor invasion, immune evasion, and angiogenesis. These researches also underscored the potential of exosomal circRNAs and associated biomarkers for non-invasive diagnosis and personalized therapeutic interventions of CCA.^13^

Subsequently, researchers turned their attention to exosome-rich bodily fluids as potential sources of diagnostic and prognostic indicators. Although circulating fluids (e.g., peripheral blood) are exosome-rich and easy to acquire, they can be interfered by exosomes derived from other diseases as well^14^; meanwhile, non-circulating fluids (e.g., bile), though harder to acquire, are supposed to better preserve the diagnostic and prognostic relevance of its correlated disease.^15^ Due to the difficulty of sample acquisition, few studies investigated the level of their target exosomal molecules in different types of bodily fluids. However, in patients with CCA combined with biliary obstruction undergoing biliary drainage, the acquisition of peripheral blood and bile samples at the same time is a “one-stone-two-birds” strategy, potentially enhancing diagnostic accuracy and prognostic insights.

Based on this idea, we innovatively analyzed the enrichment profiles of exosomal circRNAs in CCA-induced biliary obstruction (CCA-BO) and benign biliary obstruction (BBO) through liquid biopsy of two different types of bodily fluids, namely bile and serum, which are both closely related to the clinical course of CCA. We reported the discovery of a triple-circRNA complex (hsa-circ-0000367, hsa-circ-0021647, and hsa-circ-0000288), which went through in vitro and in vivo functional verification and was trained into practical diagnostic and prognostic models for potential clinical application.

## Results

### Screening of differentially enriched circRNAs in bile and serum exosomes to identify potential candidates for CCA-BO diagnosis and prognosis prediction

**Fig. 1:**
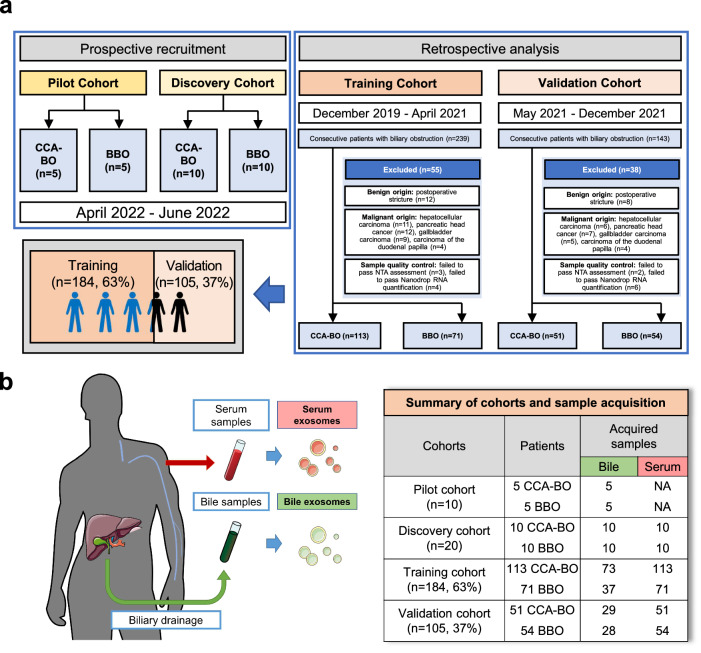
Summary of cohort recruitment and sample acquisition. **a** A pilot cohort and a discovery cohort were prospectively recruited during April 2022 and June 2022 to identify CCA-BO-specific exosomal circRNAs. A training cohort and a validation cohort were retrospectively analyzed for model establishment and verification. **b** Serum samples were acquired from all recruited patients while bile samples were acquired from patients undergoing biliary drainage, followed by exosome isolation

**Fig. 2:**
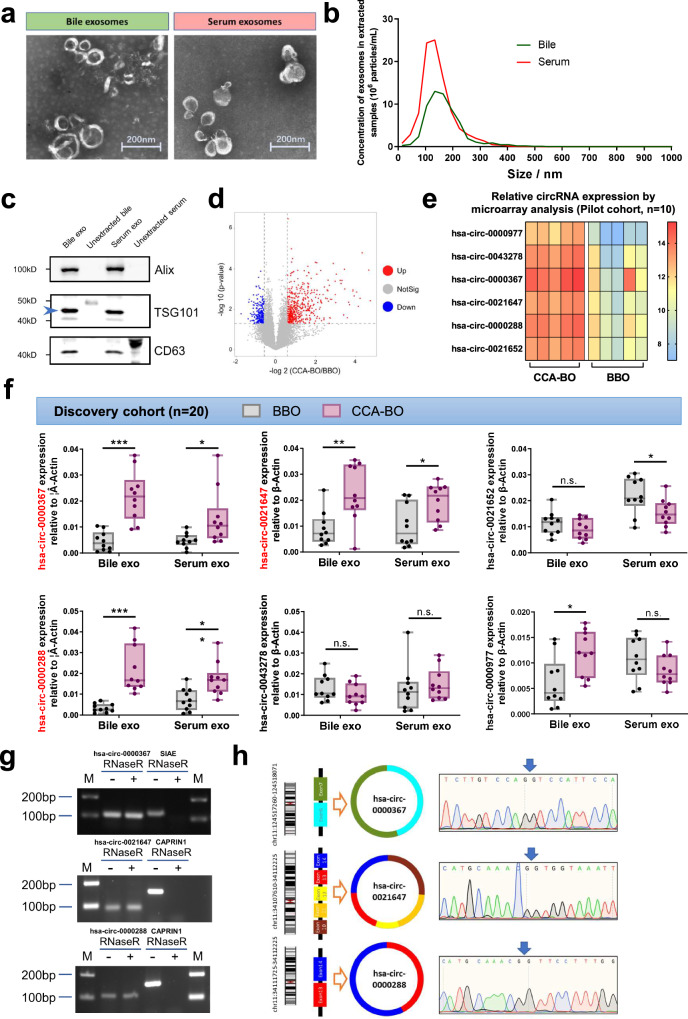
The discovery of a triple-circRNA panel in both bile and serum exosomes. The isolated exosomes were confirmed by **a** TEM, **b** NTA, and **c** immunoblotting. The main band of TSG101 was indicated with an arrowhead. **d** Volcano plot of microarray analysis revealed the pattern of differentially enriched exosomal circRNAs between CCA-BO and BBO patients. **e** Heatmap of six candidate circRNAs detected by microarray analysis. **f** Quantification of six candidate circRNAs with divergent primers in the discovery cohort. Three of them (hsa-circ-0000367, hsa-circ-0021647, and hsa-circ-0000288) were found to be upregulated in both bile and serum exosomes. **g** RNase R digestion, and **h** Sanger sequencing verified the qPCR products. *P < 0.05; **P < 0.01; ***P < 0.001; ${{\rm{n}}.{\rm{s}}.\atop }{\rm{not}}\;{\rm{significant}}$; (Mann–Whitney *U* test for continuous variables, presented as median with range and quartile)

The recruitment process of all four cohorts and sample acquisition was summarized in Fig. 1a, b. The pilot cohort consisted of ten hospitalized patients with biliary obstruction who were eventually confirmed as CCA-BO (*n* = 5) and BBO (*n* = 5) by pathology. Exosomes were isolated by differential ultracentrifugation from bile samples and were confirmed by transmission electron microscope (TEM) (Fig. 2a) and immunoblotting analysis of exosomal markers (Alix, TSG101, and CD63) (Fig. 2c). Nanoparticle tracking analysis (NTA) revealed an average diameter of 142 nm and 145 nm in the extracted exosomes of bile and serum, respectively (Fig. 2b). Microarray analysis revealed 745 differentially enriched (absolute logFC ≥1.5, *P* < 0.05) exosomal circRNAs (489 upregulated, 256 downregulated, and 12,783 conservatively expressed circRNAs) as shown in Fig. 2d and Supplementary Fig. 2a. These results revealed the active secretion of exosomal circRNAs from CCA into its microenvironment, indicating that biliary exosomal circRNAs have the potential to serve as CCA-specific biomarkers. Among the top 20 upregulated circRNAs (absolute logFC ranged from 4.75 to 3.15), 6 were selected for further investigation according to p value, documentation in circBase, origin (exonic/intronic) and in vitro pre-experiments (CCK8) as shown in Supplementary Fig. 2b. The expression profile of the 6 candidate circRNAs was visualized by heatmap (Fig. 2e).

We then went on for further screening of exosomal circRNA biomarkers. Compared with bile samples, peripheral blood has better accessibility as not all patients with obstructive jaundice undergo preoperative biliary drainage; while theoretically, biliary exosomes better reflect the status of biliary disease, as bile fluid is non-circulatory and is harder to be interfered by exosomes from non-hepatobiliary origins.^15^ To verify our hypothesis, a discovery cohort of ten CCA-BO patients and ten BBO patients were recruited to prospectively collect bile and serum samples. Divergent primers for each circRNAs were designed for quantitative real-time PCR (qPCR), and standard curve method was applied for absolute quantification. Among the six circRNA candidates, three were found to be simultaneously upregulated in bile and serum exosomes, namely hsa-circ-0000367, hsa-circ-0021647, and hsa-circ-0000288 (Fig. 2f). The circularity of the target circRNAs was verified by RNase R and dactinomycin treatment (Fig. 2g and Supplementary Fig. 2c). Sanger sequencing was also performed to verify qPCR products (Fig. 2h). These findings suggested that the selected three exosomal circRNAs have the potential to serve as CCA biomarkers in both bile and serum samples.

### Target circRNAs promote proliferation and metastasis of CCA in vitro and in vivo

**Fig. 3:**
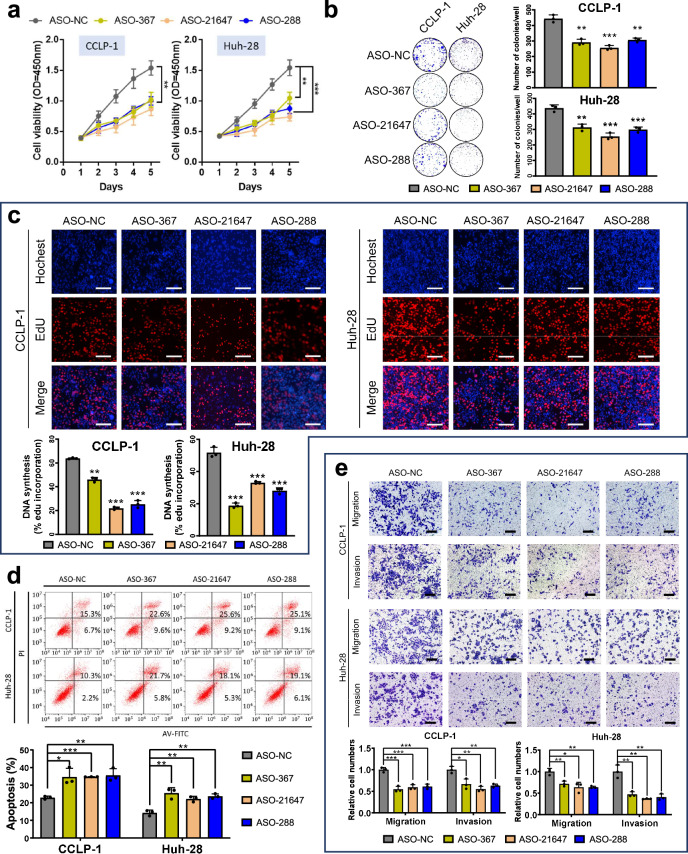
Loss-of-function assay of target circRNAs in vitro. hsa-circ-0000367, hsa-circ-0021647, and hsa-circ-0000288 were respectively knocked down by circRNA-specific ASOs (ASO-367, ASO-21647, and ASO-288) in two human primary CCA cell lines. **a** CCK8 and **b** clone formation assay and **c** Edu assay revealed short and long-term attenuation of proliferative activity in CCA cells following ASO transfection. Scale bar: 200 μm. **d** Cell apoptosis was detected by Annexin V/PI flow cytometry following ASO treatment. **e** Loss of target circRNAs attenuates CCA migration and invasion, evaluated by transwell assays. Scale bar: 500 μm. *P < 0.05; **P < 0.01; ***P < 0.001; ${{\rm{n}}.{\rm{s}}.\atop }{\rm{not}}\;{\rm{significant}}$; (two-way ANOVA with Turkey’s multiple-comparison test for curve comparison; two-tailed unpaired Student’s t test for continuous variables, presented as mean ± SD)

Before verifying the abundance of the three selected circRNAs in a larger population, we wanted to explore their functional role in the development of CCA. We used two human primary CCA cell lines (CCLP-1 and Huh-28) for in vitro verification of the three target circRNAs. Loss-of-function experiment was carried out using circRNA-specific anti-sense oligonucleotides (ASO) targeting their back-splicing sequence. The knock-down efficiency of target circRNAs was verified by qPCR, without affecting mRNA expression of their host genes (Supplementary Fig. 2d). Remarkably, ASO-mediated depletion of hsa-circ-0000367, hsa-circ-0021647, and hsa-circ-0000288 significantly reduced the viability of CCA cells in both short-term and long-term experiments (Fig. 3a, b). Edu assay revealed that the apoptotic rate was significantly elevated following depletion of each target circRNA (Fig. 3c). Annexin V/PI flow cytometry also revealed an increased proportion of cell apoptosis following ASO-mediated depletion of target circRNAs (Fig. 3d). These results suggested that silencing hsa-circ-0000367, hsa-circ-0021647, and hsa-0000288, respectively, leads to impaired CCA proliferation in vitro. We next performed a transwell assay to determine the role of target circRNAs in CCA metastasis. The results showed that the invasion and migration potential of CCA was suppressed by respectively knocking down each circRNA (Fig. 3e).

In addition, we intended to look for underlying mechanisms of these circRNA-induced malignant phenotypes. To find out whether the three circRNAs function as ceRNAs to affect downstream miRNAs and mRNAs, we constructed a circRNA–miRNA–mRNA network (Supplementary Fig. 3a). Cell adhesion molecules (CAMs) were revealed as the most significantly enriched pathway downstream, and the regulation of key molecules was verified by immunoblotting following treatment of circRNA–ASO complex in vitro (Supplementary Fig. 3b).

**Fig. 4:**
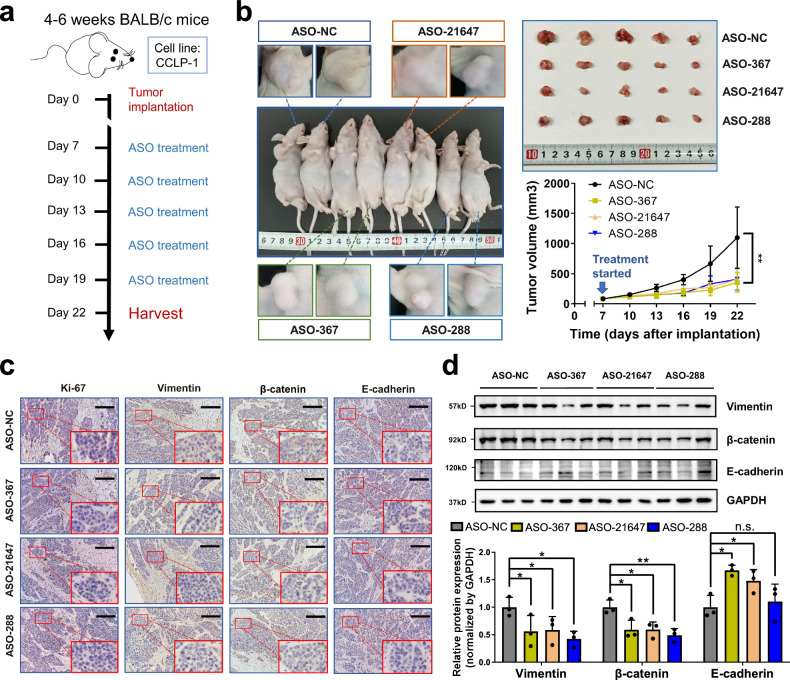
Loss of target circRNAs attenuates CCA proliferation and EMT in vivo. **a** Schematic representation of circRNA-specific ASO treatment in balb/c nude mice. CCLP-1 cells were subcutaneously injected into the right flank to establish a tumor-bearing model and randomized into ASO-NC group (*n* = 5), ASO-367 group (*n* = 5), ASO-21647 group (*n* = 5) and ASO-288 group (*n* = 5) 7 days following tumor implantation. **b** ASO treatment of each target circRNA leads to reduced tumor volume 22 days following tumor implantation. Expression of Ki-67, Vimentin, β-catenin, and E-cadherin were evaluated in harvested tumor by **c** immunohistochemistry and **d** immunoblotting. Scale bar: 100 μm. *P < 0.05; **P < 0.01; ***P < 0.001; ${{\rm{n}}.{\rm{s}}.\atop }{\rm{not}}\;{\rm{significant}}$; (two-way ANOVA with Turkey’s multiple-comparison test for curve comparison; two tail unpaired Student’s *t* test for continuous variables, presented as mean ± SD)

We then wanted to know whether these in vitro effects of the three circRNAs can be verified in vivo. CCLP-1 cells were implanted subcutaneously into BALB/c nude mice and then treated with circRNA-specific ASO by injection around tumor planting site (Fig. 4a). Based on similar initial tumor volume 1 week following implantation, circRNA-specific ASO treatment led to significant lower tumor volume compared with ASO-NC control (Fig. 4b). Tumors were harvested to investigate the expression of key biomarkers for tumor proliferation (Ki-67) and epithelial-to-mesenchymal transition (EMT) (markers: E-cadherin, β-catenin, and Vimentin), since these markers are known to be associated with biliary oncogenesis.^16,17^ Immunochemistry revealed decreased expression of Ki-67, β-catenin, Vimentin, and increased expression of E-cadherin in circRNA-specific ASO-treated groups, which was further validated by immunoblotting (Fig. 4c, d).

These in vitro and in vivo findings indicated that these three circRNAs may support the progression of CCA, thereby potentially influencing the prognosis of patients who have undergone treatment.

### Construction and verification of target circRNA-based diagnostic model

To establish a diagnostic and prognostic model intended for clinical use, we then investigated the expression profile of the 3 circRNAs in both bile and serum exosomes based on a larger population. A training cohort (71 BBOs and 113 CCA-BOs) was retrospectively analyzed to collect bile and serum samples along with the clinicopathological features of the patients. Before verifying the abundance of target circRNAs, we examined the performance of lab tests currently used in CCA-BO diagnosis. We first conducted a baseline comparison between the benign and malignant groups (Table 1). The results showed that there was a significant difference between the BBO group and CCA-BO group in terms of CA19-9, CEA, CA125, TB, DB, DB/TB ratio, ALT, AST, and ALB. Next, the diagnostic potential of these biomarkers was evaluated by area under the receiver operating characteristic curves (AUROC). We used previously described criteria to estimate the diagnostic power of these biomarkers according to AUROC: an AUROC of 0.5 suggests no discrimination, 0.7–0.8 is considered acceptable, 0.8–0.9 is considered excellent, and more than 0.9 is considered outstanding.^18^ As expected, CA19-9 was revealed as the most eligible biomarker for CCA-BO diagnosis (AUROC = 0.759), which is merely considered acceptable. Other potentially useful biomarkers were DB (AUROC = 0.714), DB/TB ratio (AUROC = 0.711), CEA (AUROC = 0.707), TB (AUROC = 0.706), and CA125 (AUROC = 0.699) (Supplementary Fig. 4). These findings suggested that the currently used biomarkers for CCA-BO diagnosis is still not satisfactory.

**Table 1:** Baseline characteristics of patients in the training cohort and validation cohort to establish a diagnostic model

Covariates	Training cohort			Validation cohort		
	BBO (*n* = 71)	CCA-BO (*n* = 113)	*P* value	BBO (*n* = 54)	CCA-BO (*n* = 51)	*P* value
Sex			0.251			0.073
Male	41 (57.7%)	76 (67.3%)		29 (53.7%)	37 (73.5%)	
Female	30 (42.3%)	37 (32.7%)		25 (46.3%)	14 (27.5%)	
Age	52 (41, 59)	59 (51, 63)	<0.001	54 (43, 61)	62 (52, 66)	0.002
CCA subtypes			NA			NA
iCCA involving the hepatic hilus	NA	24 (21.2%)		NA	10 (19.6%)	
pCCA	NA	53 (46.9%)		NA	26 (51.0%)	
dCCA	NA	36 (31.9%)		NA	15 (29.4%)	
Benign etiologies			NA			NA
Bile duct stones	42 (59.2%)	NA		32 (59.3%)	NA	
Inflammation	29 (40.8%)	NA		22 (40.7%)	NA	
CA19-9	16.8 [7.1, 35.3]	117.0 [21.3, 537.0]	<0.001	15.8 [6.8, 57.7]	260.0 [54.5, 745.0]	<0.001
CEA	1.8 [1.5, 2.9]	3.4 [2.0, 7.3]	<0.001	1.9 [1.3, 3.1]	2.7 [1.8, 4.7]	0.003
CA125	13.5 [9.7, 17.4]	21.2 [11.9, 40.7]	0.002	11.9 [9.7, 18.8]	23.1 [13.8, 59.6]	0.004
AFP	3.3 [2.4, 4.1]	3.8 [2.3, 7.0]	0.125	2.6 [2.0, 3.2]	2.7 [1.7, 3.5]	0.665
TB	49.9 [36.6, 104.6]	131.0 [49.6, 252.5]	<0.001	29.4 [21.7, 49.8]	188.4 [103.5, 307.5]	<0.001
DB	31.8 [21.9, 68.6]	113.1 [40.1, 214.6]	<0.001	19.7 [13.9, 37.8]	157.0 [89.1, 249.2]	<0.001
DB/TB	0.64 [0.57, 0.73]	0.82 [0.75, 0.89]	<0.001	0.68 [0.63, 0.82]	0.82 [0.77, 0.87]	0.004
ALT	45 [26, 81]	67 [41, 120]	<0.001	46 [26, 105]	86 [53, 128]	0.023
AST	44 [29, 67]	64 [41, 103]	<0.001	47 [29, 91]	69 [53, 119]	0.018
ALB	42 (5.4)	39 (4.3)	<0.001	41 (10.4)	36 (4.4)	0.003

Continuous variables with normal distribution are presented as mean value (SD) while others are presented as median [IQR]. Categorical variables are presented as frequency (percentage)

**Fig. 5:**
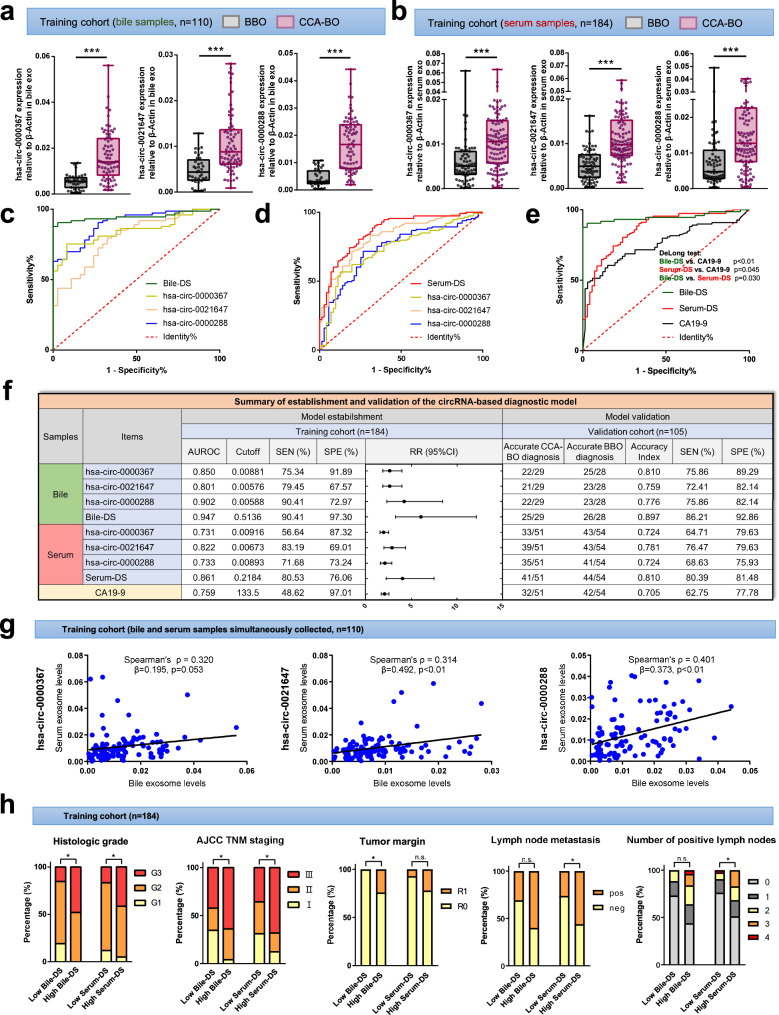
Establishment and validation of diagnostic models. The abundance of hsa-circ-0000367, hsa-circ-0021647, and hsa-circ-0000288 in **a** bile and **b** serum exosomes were measured by qPCR in the training cohort. ROC curves of **c** bile and **d** serum circRNA signatures (Bile-DS and Serum-DS) were illustrated. **e** The diagnostic power of Bile-DS, Serum-DS and CA19-9 was compared. **f** Summary of the established diagnostic models and their verification in the validation cohort. Cutoff values were determined by optimal Youden’s index. **g** The abundance of circRNAs in bile exosomes and serum counterparts showed correlation with each other. **h** Patients in the training cohort were grouped by high/low diagnostic scores and compared of clinical outcomes. *P < 0.05; **P < 0.01; ***P < 0.001; ${{\rm{n}}.{\rm{s}}.\atop }{\rm{not}}\,{\rm{significant}}$; (Mann–Whitney *U* test for continuous variables, presented as median with range and quartile; Pearson’s chi-squared test for categorical variables)

Next, we quantified the three circRNAs in the training cohort and found that, consistent with the discovery cohort, hsa-circ-0000367, hsa-circ-0021647, and hsa-0000288 were significantly upregulated in CCA-BO group in both bile and serum exosomes (Fig. 5a, b). Based on the qPCR data measured by absolute quantification, we first evaluated the diagnostic potential of each circRNA, and then established two pooled models via logistic regression:

$$\begin{array}{l}{Bile}\,{diagnostic}\,{score}\,({Bile}-{DS})=-4.829+219.749\times {{Exp}}_{{hsa}{{-}}{circ}{{-}}0000367}+210.340 \\\qquad\qquad\qquad\qquad\qquad\qquad\qquad\qquad\times{Exp}_{{{hsa}}-{{circ}{{-}}0021647}}+254.645\times {{Exp}}_{{hsa}{{-}}{circ}{{-}}0000288}\end{array}$$

$$\begin{array}{l}{Serum\; diagnostic\; score}\,({Serum}-{DS})=-2.945+35.448\times {{Exp}}_{{hsa-circ-}0000367}+290.995\\\qquad\qquad\qquad\qquad\qquad\qquad\qquad\qquad\qquad\times {{Exp}}_{{hsa-circ-}0021647}+72.636\times {{Exp}}_{{hsa-circ-}0000288}\end{array}$$

The goodness-of-fit was measured by component plus residual plots (Supplementary Fig. 7b). We also verified the independency of Bile-DS and Serum-DS by performing Spearman’s correlation analysis with currently used clinical indices. The results showed that Bile-DS and Serum-DS were independent from serum CA19-9, CEA, CA125, TB, DB, ALT, AST, and ALB levels, at the same time had moderate to strong correlation (Spearman’s ρ = 0.536, *P* < 0.001) with each other (Supplementary Figs. 6 and 7a). The diagnostic accuracy of each circRNA and their pooled models were evaluated by AUROC. The results showed that, compared with individual circRNAs, the pooled model had better diagnostic accuracy in bile (AUROC 0.947 vs. 0.850 or 0.801 or 0.902) and in serum (AUROC 0.861 vs. 0.731 or 0.822 or 0.733) (Fig. 5c, d). Meanwhile, we compared the diagnostic accuracy of Bile-DS, Serum-DS and CA19-9, revealing Bile-DS as the most accurate index (AUROC = 0.947; DeLong test: Bile-DS vs. CA19-9 p < 0.01, Bile-DS vs. Serum-DS *P* = 0.030), followed by Serum-DS (AUROC = 0.861; DeLong test: Serum-DS vs. CA19-9 *P* = 0.045) and CA19-9 (AUROC = 0.759) (Fig. 5e). These results suggested that this bile/serum exosomal circRNA panel may serve as a novel diagnostic tool in patients with CCA combined with biliary obstruction. For further verification, a validation cohort (54 BBOs and 51 CCA-BOs) was retrospectively analyzed and assayed using the same methods. Using the cutoff value determined by the training cohort, we verified the diagnostic performance of our established models (Fig. 5f). The accuracy index (ACC) of Bile-DS reached 0.897, followed by Serum-DS (ACC = 0.810) and CA19-9 (ACC = 0.705). Furthermore, we performed a propensity score match analysis to adjust the baseline of the CCA-BO group and the BBO group, in order to simulate more challenging application scenarios. The results showed that, Bile-DS (AUROC = 0.921, RR = 8.40 [95% CI, 2.78–30.30]) and Serum-DS (AUROC = 0.826, RR = 2.33 [95% CI, 1.63–3.63]) remained to be powerful diagnostic indices for CCA-BO in the training cohort after adjustment of clinicopathologic features including Sex, Age, CA19-9, CEA, CA125, TB, DB, ALT, AST, and ALB (Supplementary Figs. 8 and 9). Similar results were noted in the validation cohort (Bile-DS: ACC = 0.852; Serum-DS: ACC = 0.792) (Supplementary Figs. 10 and 11). These results suggested that our diagnostic model based on the triple-circRNA combination could robustly distinguish CCA-BO from benign origins.

Few studies involved detection of target ncRNAs in different types of bodily fluids, and their correlation remained unnoticed. Therefore, we performed a correlation analysis between the abundance of circRNAs in bile exosomes and their serum counterparts, revealing a moderate correlation (Fig. 5g and Supplementary Fig. 5). Based on these findings, we proposed that CCA-derived exosomal circRNAs can be detected simultaneously by bile and serum liquid biopsy.

Given that the three circRNAs had positive in vitro and in vivo findings in terms of CCA progression, we assumed that they might be related with some clinicopathological features of CCA-BO. To test this hypothesis, we divided CCA-BO patients in the training cohort into two groups based on their Bile-DS and Serum-DS levels, and compared postoperative outcomes between the two groups. The results showed significant differences in histologic grading, R0 resection rate, lymph node status and AJCC TNM staging (Fig. 5h and Supplementary Table 1). These findings suggested that the three circRNAs may also serve as prognostic indicators in CCA-BO patients undergoing surgical treatment.

### Construction and verification of target circRNA-based prognostic model

**Fig. 6:**
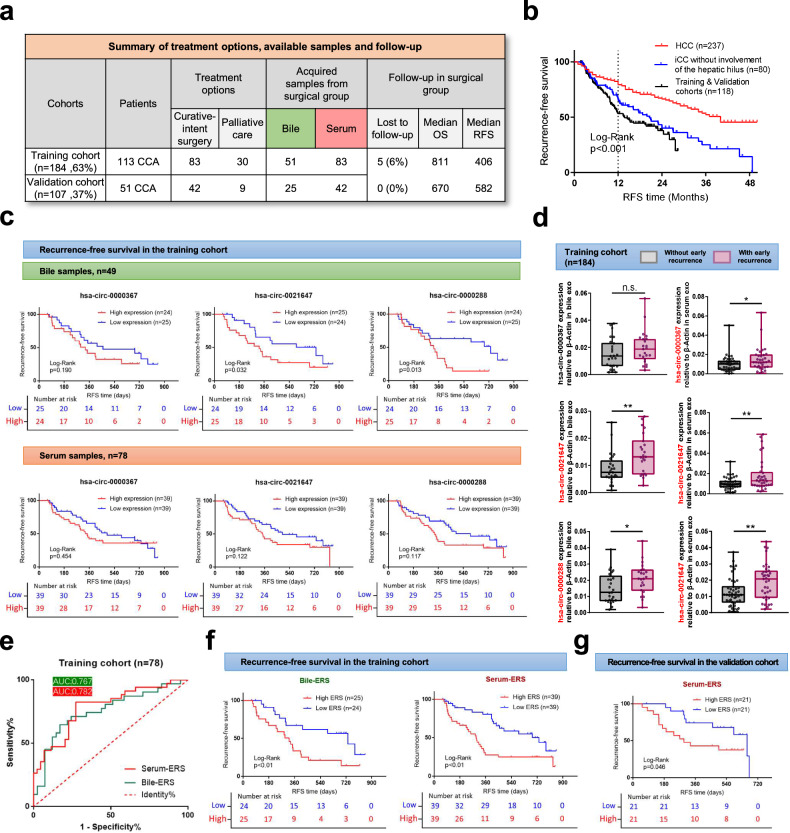
Establishment and verification of early recurrence monitoring models. **a** Patients who underwent curative-intent surgery in the training cohort and validation cohort were followed up. **b** Recurrence patterns were compared in patients with HCC (*n* = 237), iCCA without involving the hepatic hilus (*n* = 80) and the training and validation cohorts (*n* = 118) from a single center to determine the best cutoff for early recurrence of CCA-BO patients. **c** Patients were grouped by their circRNA abundance to compare the recurrence-free survival. **d** Patients were grouped by the presence of early recurrence (1 year) to compare circRNA abundance. **e** ROC curves of bile and serum circRNA signatures (Bile-ERS and Serum-ERS) were illustrated. Patients were grouped by their Bile-ERS/Serum-ERS levels to compare the recurrence-free survival in (**f**) the training cohort and **g** the validation cohort. *P < 0.05; **P < 0.01; ***P < 0.001; ${{\rm{n}}.{\rm{s}}.\atop }{\rm{not}}\,{\rm{significant}}$; (Mann–Whitney *U* test for continuous variables, presented as median with range and quartile; Kaplan–Meier analysis for survival data, Log-Rank test for curve comparison)

To establish a prognostic model, CCA-BO patients who underwent curative-intent surgery in the training cohort (*n* = 83) and the validation cohort (*n* = 42) were followed up (Fig. 6a). Baseline characteristics of these surgical patients were summarized (Supplementary Table 2). We first performed a survival analysis and found indistinctive correlations between bile/serum exosomal level of each circRNA and overall survival of CCA-BO patients in both training and validation cohorts (Supplementary Figs. 12 and 13). Similarly, the correlation between each circRNA and recurrence-free survival (RFS) of CCA-BO patients was noted to be significant in only a few instances (Fig. 6c and Supplementary Fig. 14a). Considering the high invasiveness of CCA, we hypothesized that our target circRNAs might have better prediction value within a certain postoperative period. Previous research found that CCA tend to have early recurrence following curative-intent surgery, defined by a cutoff of 12 or 24 months following resection.^19^ Using a retrospective dataset from our medical center, we compared the recurrence patterns of patients with HCC and iCCA (without involvement of the hepatic hilus) along with patients from our cohorts (Fig. 6b). The results showed that, compared with HCC patients undergoing surgical resection, patients with CCA tend to have poorer RFS (Log-Rank *P* < 0.001). By a cutoff of 1 year, the RFS proportion in the three groups were 79.3%, 66.1%, and 53.3%, respectively. We then compared the abundance of each circRNA by dividing the patients using an early recurrence cutoff of 1 year. Remarkably, hsa-circ-0021647 and hsa-0000288 were significantly elevated in bile exosomes of patients with early recurrence, while similar outcome was noticed in all three serum exosomal circRNAs (Fig. 6d). The predictive effect of individual circRNAs was unremarkable (Supplementary Fig. 15). We then conducted a multivariate stepwise logistic regression analysis to determine target circRNAs to be included in the early recurrence model (Supplementary Fig. 16a). Early recurrence scores (ERS) were calculated by logistic regression analysis:

$${Bile\; early\; recurrence\; score}\,({Bile}-{ERS})=-2.522+116.399\times {{Exp}}_{{hsa-circ-}0021647}+61.133\times {{Exp}}_{{hsa-circ-}0000288}$$

$${Serum\; early\; recurrence\; score}\,({Serum}-{ERS})=-2.945+35.448\times {{Exp}}_{{hsa-circ-}0000367}+290.995\times {{Exp}}_{{hsa-circ-}0021647}+72.636\times {{Exp}}_{{hsa-circ-}0000288}$$

Goodness-of-fit of the models was measured by component plus residual plots (Supplementary Fig. 16b). The prognostic power of the models was verified by AUROC (training cohort, Bile-ERS: AUROC = 0.767; Serum-ERS: AUROC = 0.782; validation cohort, Bile-ERS: AUROC = 0.851; Serum-ERS: AUROC = 0.759) (Fig. 6e and Supplementary Fig. 16c). We then divided the patients in the training cohort into a high ERS group and a low ERS group, and found that both Bile-ERS and Serum-ERS were closely related to RFS (Fig. 6f). This was followed by verification in the validation cohort (Fig. 6g and Supplementary Fig. 14b). These results suggested that this recurrence monitoring model based on ERS is predictive of early recurrence of CCA and has high translational potential.

**Fig. 7:**
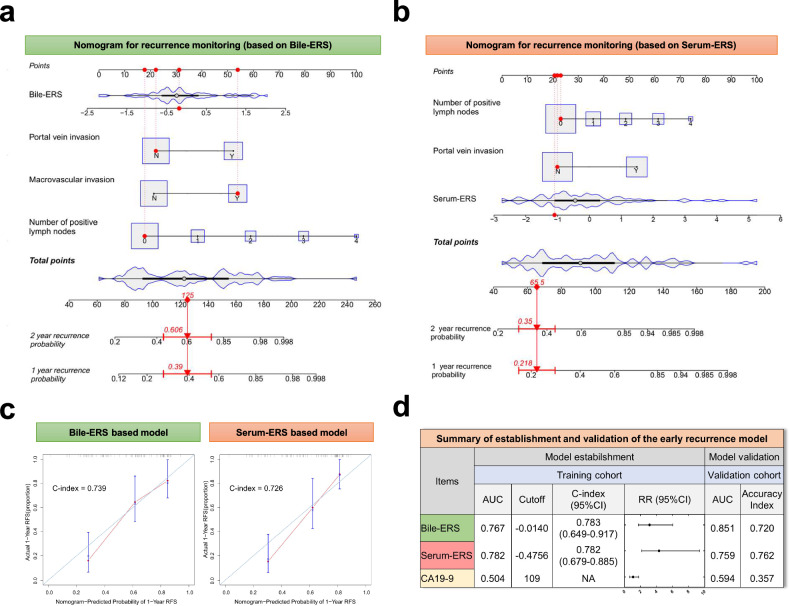
Establishment of nomograms for CCA recurrence monitoring. **a** Bile-DS and **b** Serum-DS were trained into nomograms along with other prognostic predictors revealed by COX-PH analysis. **c** The prediction accuracy of the nomograms was verified by calibration curves. The 45-degree reference line represents an ideal nomogram. **d** Summary of the establishment and verification of CCA recurrence monitoring signatures

Based on this circRNA model aimed at predicting early recurrence of CCA, we sought for practical tools for the calculation of overall recurrence risk. A multivariate COX regression analysis was conducted to identify potential indicators for postoperative CCA recurrence. Bile-ERS and Serum-ERS were respectively analyzed with other covariates, including histologic grade, AJCC TNM staging, CCA subtype, tumor margin, preoperative biliary drainage, tumor diameter, lymph node status, macrovascular invasion, portal vein invasion and microvascular invasion. Schoenfeld’s global test was applied to test the proportional hazards assumption (Supplementary Figs. 17 and 18). The number of positive lymph nodes, portal vein invasion and macrovascular invasion were revealed as risk factors along with Bile-ERS; while number of positive lymph nodes and portal vein invasion were revealed as risk factors along with Serum-ERS (Supplementary Fig. 19). Nomograms based on the COX-PH model were subsequently established (Fig. 7a, b). The C-index of Bile-ERS/Serum-ERS-based models were 0.739 (95% CI, 0.723–0.755) and 0.726 (95% CI, 0.709–0.743), respectively. Calibration plots showed the close agreement between the predicted RFS and the actual status (Fig. 7c). The established recurrence monitoring models were verified by the validation cohort: the AUROC of Bile-ERS and Serum-ERS were 0.851 and 0.759, respectively, and the prognostic accuracy using cutoff values defined by the training cohort were 0.720 and 0.762, respectively (Fig. 7d). In summary, these findings strongly suggested that our circRNA-based prognostic models are robust tools for potential clinical application, and a schematic workflow on how to choose between detection strategies based on bile and serum was provided (Supplementary Fig. 20).

## Discussion

In the past few years, novel biomarkers emerged to support non-invasive and more precise diagnosis and prognosis prediction in various types of malignancies. However, the laboratory diagnosis and prognosis prediction of CCA still remains a challenge. Our team conducted a single-center cohort study to identify exosomal circRNAs which serve as diagnostic and prognostic indicators for patients with CCA combined with obstructive jaundice. A triple-circRNA panel was discovered via high-throughput sequencing following stepwise screening and verification in three cohorts. In vitro and in vivo experiments were also conducted to cross-validate the CCA-promotive function of the three circRNAs. Innovatively, we discovered this circRNA panel in two different types of bodily fluids, namely bile and serum, and verified that they could both serve as promising biomarkers in different application scenarios. Our research was inspired by the fact that the biomarkers for CCA diagnosis and prognosis prediction are lacking. CA19-9, as the clinically applied serum biomarker for CCA diagnosis, is limited in several aspects: (1) its high false-positive rate in patients complicated with obstructive jaundice^8^; (2) it’s not a CCA-specific biomarker, with easily altered circulating levels by non-hepatobiliary diseases; (3) its prognostic value for CCA remains controversial.^20^ Therefore, the goal of this study was to identify novel biomarkers which are CCA-specific, perform effectively in patients with obstructive jaundice, and better have prognostic value as well.

The first key issue was to decide which type of biomarkers to be detected. Exosomes have been regarded as promising biomarkers in the diagnosis and prognosis prediction of malignancies, since they carry various types of cargoes from their tissue of origin. One of the highlighted exosomal contents is circRNA, which is a type of unusually stable ncRNA enriched in tumor-derived exosomes. Many studies reported the identification of tumor-predictive exosomal circRNAs, including in liver cancers, colon cancers, glioma, etc.^10,21,22^ However, few sample-based clinical studies discussed the diagnostic and prognostic value of exosomal circRNA in biliary malignancies.^11,12^ Hence, we selected exosomal circRNAs as the object of our study. Based on the preliminary results of microarray analysis, we identified a CCA-related triple-circRNA combination via stepwise validation in three cohorts. We established an interpreter-free diagnostic system by pooling the effect of individual circRNAs together. Notably, these three circRNAs were not only found to be upregulated in CCA-BO patients, but also contributes to the progression of CCA in vitro and in vivo. Based on these findings, we further analyzed the correlation between postoperative prognosis and the abundance of target circRNAs. Although not correlated with overall survival of patients with CCA undergoing curative-intent surgery, this circRNA panel was found to be closely related to early recurrence of CCA, defined as one year following tumor resection. We thereby established practical prognostic tools based on logistic regression model to predict early recurrence; and Cox PH model-based nomograms to monitor the overall risk of CCA recurrence. To sum up, the triple-circRNA signature was verified as a robust predictor of CCA-BO and CCA recurrence following curative-intent surgery, and has high potential to be translated into clinics. The second key issue was to choose between different types of clinical samples. Undoubtedly, peripheral blood, which is easy to access by minimally invasive approaches, serves as the most widely accepted material for exosome isolation. Many studies have reported discovery of novel tumor biomarkers in serum or plasma-derived exosomes. Specified to biliary malignancies, Lapitz et al. reported isolation of proteins from extracellular vesicles in serum, from which biomarkers for CCA diagnosis were identified.^23^ Wang et al. reported the discovery of two plasma-derived circRNAs in a small iCC cohort.^11^ However, as a circulating fluid, peripheral blood does not specifically reflect the status of the biliary system, and can be altered by other diseases.^14^ Therefore, we turned our attention to bile, which is a non-circulating fluid and thereby has closer relationship with the biliary system in theory. This idea is well supported by recent studies. Hoshino et al. reported collection of bile duct fluid specimen to establish biliary cancer-related proteomic profile in extracellular vesicles.^24^ Similarly, Severino et al. reported the diagnostic value of biliary extracellular vesicle concentration in malignant biliary stenosis.^15^ Taken these together, we intended to conduct our research based on both serum and bile samples. The scope of our study was limited to patients with CCA combined with biliary obstruction, as many of them undergo biliary drainage as part of their treatment. Subsequently, acquisition of bile samples in these patients can be achieved through a single invasive procedure.

The analysis of preoperatively collected bile and serum samples demonstrated that the exosomal triple-circRNA signature performed better than CA19-9 alone for predicting the presence of CCA. Interestingly, either bile or serum-derived exosomal circRNAs alone demonstrated noninferior value than CA19-9 in discriminating CCA from benign origin. More importantly, although CA19-9 performed better in terms of specificity, exosomal circRNAs showed higher sensitivity, which is quite crucial for early surveillance of the disease. Combining the advantages of the three circRNAs, both bile and serum-derived circRNA signatures demonstrated superiority in detection of CCA when compared to CA19-9 alone. However, future direct head-to-head comparisons in clinical studies are required to establish superiority of the circRNA signature for early screening of CCA.

Our results also suggested that there was a higher diagnostic efficacy in the bile exosome-based signature compared with the serum-based counterpart. Our hypothesis is that, according to the physiological nature of CCA, there is a significantly higher enrichment of CCA-derived exosomes in bile than in blood. However, the acquisition of bile samples involves invasive procedures. In clinical practice, not all patients suspected with malignant biliary obstruction undergo preoperative biliary drainage, which limits the accessibility of bile samples. On the other hand, serum samples are easier to acquire by gathering peripheral blood. Our results also demonstrated moderate to strong correlation between the same circRNA levels in bile and serum exosomes. Therefore, exosomal circRNAs in bile and serum samples should not be considered as two independent sets of biomarkers, instead they are two types of detection strategies to be chosen according to practical needs (Supplementary Fig. 20).

Targeted therapy for cholangiocarcinoma is still in its infancy in clinical practice, making the development of effective diagnostic and prognostic tools crucial for improving treatment outcomes. In this context, our study endeavors to contribute to the development of such tools. Several limitations of this study should be acknowledged, yet these constraints may also serve as guideposts for future researches. This study consists of four cohorts, among which two main cohorts for model construction and validation were retrospectively analyzed, accounting for potential bias. RNA sequencing data on bile exosomes is lacking, which can provide more in-depth insights to the relationship of biliary disease and exosomal circRNAs. Additionally, the cancer-promoting mechanism of the target circRNAs has not been fully elucidated, as this research mainly focused on clinical translational potential. Moreover, this is a single-centered study with the majority of recruited patients from East Asia, and the influence of geographic variation was not discussed. Despite these limitations, this study is by now the most comprehensive study to evaluate the potential clinical utility of exosomal circRNAs in the diagnosis and prognosis prediction of CCA, and involved analysis of two different types of bodily fluids for liquid biopsy.

In conclusion, this study established an exosomal circRNA signature based on serum and biliary liquid biopsy to create a novel system in the identification and recurrence monitoring of CCA. The proposed circRNA signature may assist the diagnosis of CCA and CCA-related obstructive jaundice, meanwhile may provide valuable information in the clinical decision-making process concerning the treatment of CCA.

## Materials and methods

### Study population

This study involves observational cohorts from a single center (the Department of Biliary Surgery, West China Hospital, Chengdu, China) and was registered in the Chinese Clinical Trial Registry (ChiCTR2300069863, available at http://www.chictr.org.cn). The study protocol was approved by the Ethics Committee of Biomedical Research, West China Hospital of Sichuan University. All participants provided written informed consent before enrollment, and the study was conducted in accordance with Good Clinical Practice guidelines and the Declaration of Helsinki. The workflow of this study was graphically demonstrated (Supplementary Fig. 1). In short, patients with biliary obstruction were recruited and subsequently grouped by malignant obstruction and benign obstruction following pathological diagnosis (confirmed either by postoperative pathology, percutaneous biopsy or ERCP-guided biopsy). To minimize confounding, only patients with CCA were included in the malignant obstruction group (namely iCCA involving the hepatic hilus, pCCA, and dCCA), while patients confirmed as benign biliary obstruction with either bile duct stone or inflammatory etiology were included in the BBO group (inclusion and exclusion criteria shown in Fig. 1a). A pilot cohort (5 CCA-BO vs. 5 BBO) and a discovery cohort (10 CCA-BO vs. 10 BBO) were recruited prospectively between April 2022 and June 2022 for exploratory research. In the training cohort, biospecimen of 239 consecutive patients diagnosed with obstructive jaundice from December 2019 to April 2021 were retrospectively analyzed to identify 184 eligible patients (63% of training and validation cohorts, 113 CCA-BO and 71 BBO); while in the validation cohort, biospecimen of 143 consecutive patients diagnosed with obstructive jaundice from May 2021 to December 2021 were retrospectively analyzed to identify 105 eligible patients (37% of of training and validation cohorts, 51 CCA-BO and 54 BBO). The whole recruitment workflow is shown in Fig. 1a.

### Sample collection and exosome isolation

Detailed methods for sample collection and exosome isolation were described in Supplementary Methods. In short, samples were collected prospectively in the pilot cohort and discovery cohort while retrospectively attained from the Biobank of West China Hospital in the training cohort and validation cohort. The collection of biospecimen was conducted according to a set of pre-determined protocols for quality control. Of note, samples were collected preoperatively in those patients undergoing surgical treatment to avoid bias. Serum samples were attained from all recruited patients, while bile samples were obtained from patients undergoing biliary drainage, collected during ERCP or PTBD and immediately stored at −80 °C until further processing and testing. The isolation of exosomes was conducted via differential ultracentrifugation, verified by Transmission Electron Microscope (TEM), Nanoparticle Tracking Analysis (NTA), and exosomal markers.^25^ The isolation process was proceeded within 6 h. Isolated exosomes were stored at −80 °C and documented before further processing.

### Clinicopathological characteristics and postoperative outcomes

As described in “study population”, all recruited patients underwent at least one pathological test to confirm CCA or benign origin diagnosis. Clinical assessments including general demographic information, CA19-9 levels, liver function tests and imaging diagnosis were obtained. TNM staging was classified postoperatively according to the American Joint Committee on Cancer (AJCC) staging for cholangiocarcinoma.^26^ Early recurrence of CCA was defined by a cutoff of 1 year following surgical resection after comprehensive consideration of previous researches as well as our own data in terms of iCCA, pCCA and dCCA recurrence.^19,27–29^

After discharge, patients were followed every 1–3 months in the first year and every 3 months thereafter. Blood tests, liver function tests, tumor marker tests as well as abdominal ultrasonography were routinely measured during each follow-up session. CT or MRI was performed as soon as the recurrence was suspected in ultrasound imaging. Telephone and online interviews were conducted in order to ensure the health condition of patients. Clinicopathological data was de-identified before admitting into the database.

### Statistical analysis

This study involved statistical evaluation of diagnostic performance and survival analysis concerning a group of novel exosomal biomarkers, as was instructed by reference guides.^30,31^ In general, an interpreter-free diagnostic system was constructed and validated using linear regression-based algorithms by combining and pooling independent biomarkers. Following precursive survival analysis using the Kaplan–Meier method, Cox proportional hazards (PH) model was applied to establish a recurrence monitoring system after checking the PH assumption. The more detailed methodology is available in Supplementary Materials.

## Supplementary information

Supplementary files

## Data Availability

Results of the microarray analysis have been uploaded to the GEO database (ID: GSE228477, available at https://www.ncbi.nlm.nih.gov/gds). Upon reasonable request, the data supporting the findings of this study will be provided to the scientific community.
