# Supplementary material for: Cholangiocarcinoma combined with biliary obstruction: an exosomal circRNA signature for diagnosis and early recurrence monitoring
Source: Signal Transduct Target Ther. 2024 May 3;9:107. doi: 10.1038/s41392-024-01814-3 (PMC11636852; doi:10.1038/s41392-024-01814-3)
Supplement: Supplementary file 1 — Supplementary files [file 41392_2024_1814_MOESM1_ESM.docx]

Supplementary Materials for

**Cholangiocarcinoma combined with biliary obstruction: an exosomal circRNA signature for diagnosis and early recurrence monitoring**

Ningyuan Wen^#1,2^, Dingzhong Peng^#1,2^, Xianze Xiong^#1,2^, Geng Liu^1,2^, Guilin Nie^1,2^, Yaoqun Wang^1,2^, Jianrong Xu^1,2^, Shaofeng Wang^1,2^, Sishu Yang^1,2^, Yuan Tian^1,2^, Bei Li*^1,2^, Jiong Lu*^1,2^, Nansheng Cheng*^1,2^

Correspondence to: nanshengcheng@yeah.net

lujiong@scu.edu.cn

libei@scu.edu.cn

**This PDF file includes:**Supplementary Materials and Methods
Supplementary Figures and Tables
Figures. S1 to S14
Tables S1 to S2
References for Supplementary Materials

**Supplementary Materials and Methods**

**Exosome isolation**

Our protocol for exosome isolation is a modified version of differential ultracentrifugation approach reported by Thery et al.^1^ Samples were thawed at 4°C overnight and then sequentially centrifuged three times (300*g*, 4°C, 10 minutes/ 2000*g*, 4°C, 10 minutes/10000*g*, 4°C, 30 minutes) to remove cells and debris. Ultracentrifugation was conducted with Optima XPN-100 and Optima MAX-XP (Beckman Coulter, USA). Bile sample (20 mL) and serum sample (2 mL) were both diluted with D-PBS at a 1:1 ratio to reduce viscosity, and centrifuged (at 12000*g* and 30 minutes) at 4°C to further remove cellular debris. Next, supernatants were filtered with a 0.22-μm filter and subsequently ultracentrifuged twice (at 120000*g*, 120 minutes, 4°C) to pellet the exosomes. Collected pellets were resuspended in 50 µl of D-PBS and stored at - 80ºC for further experiments.

**Protein Immunoblotting**

Cultured cells were lysed in RIPA buffer (Beyotime, China) and protease inhibitor cocktail (Beyotime, China) at a concentration of 10 mg/mL; exosomal pellets were lysed in a 1:1 ratio with RIPA buffer; harvested xenograft tissue was lysed for 40 min on ice in buffer containing 50 mmol/L NaCl, 0.5% Triton X-100, 50 mmol/L Tris-HCl (pH 7.4), 25 mmol/L NaF, 20 mmol/L EDTA, 1 mmol/L DTT, 1 mmol/L Na3VO4, and protease inhibitors at a concentration of 10 mg/mL. The protein concentration was quantified with a BCA kit (Thermo, USA). The protein samples were subjected to 10% SDS-PAGE and transferred to a PVDF membrane (Millipore, USA). The membrane was blocked for 1 h in 5% skim milk at room temperature before an incubation at 4°C overnight with primary antibody diluted as follows: (1) rabbit monoclonal anti-TSG101 (ab125011; Abcam, UK) 1:500; (2) rabbit monoclonal anti-CD81 (ab109201; Abcam, UK) 1:500; (3) mouse monoclonal anti-ALIX (sc-271975; Santa Cruz, USA) 1:500; and (4) rabbit monoclonal anti-N-cadherin, anti-E-cadherin, anti-β-catenin, anti-vimentin (WanleiBio, China) 1:500. After washing with PBS-T, membranes were further incubated with an anti-rabbit or anti-mouse secondary antibody (1:5000, ZAGB-Bio, China) and visualized in ChemiDoc imager system (Bio-Rad, USA).

**Transmission electron microscopy (TEM)**

A total of 10 μL of exosomal pellets was loaded directly onto glow-discharged holey carbon grids and incubated at room temperature for 5 minutes. Then samples were fixed with 2% paraformaldehyde at room temperature for 5 minutes, and rinsed thrice in PBS buffer. Next, samples were negatively stained in 4% uranyl acetate for 1 minutes. Afterwards, stain was blotted dry by draining excess liquid with filter paper, and samples were left to air dry for a few minutes. Samples were examined under the transmission electron microscope (Tecnai G2 Spirit Bio TWIN, FEI, USA) at an accelerating voltage of 80 Kv.

**Nanoparticle tracking analysis (NTA)**

# To measure size distribution and concentration of bile/ serum exosomal pellets, samples were processed by ZetaView^®^ x30 (Particle Metrix, German), and collected data was analyzed using the nanoparticle tracking analysis (NTA) software (Particle Metrix, German). The recording camera level was set to 9 and the processed threshold level of detection to 5, respectively. Exosomal pellets were diluted with pre-filtered (0.22 μm) D-PBS at different ratios so as to match the optimal range of the instrument.

**Microarray analysis**

A Human circRNA Array (Arraystar, Kangchen, China) analysis specifically for human circular RNAs splicing sites was used with 6 bile-derived exosomes samples (including 3 CCA patients and 3 BDS patients). To eliminate the linear RNA and enrich for circular RNA, the total RNA underwent treatment with RNase R (Epicentre, USA). The random priming method was used to amplify and transcribe the purified RNA samples into fluorescent cDNA using (Arraystar Super RNA Labeling Kit; Arraystar). Subsequently, the labeled cRNAs were hybridized onto the Arraystar Human circRNA Array (8x15K, Arraystar) and incubated at 65°C for 17 h. Finally, the slides were washed and scanned with an Agilent Scanner G2505C (Agilent, USA). The acquired array images were analyzed with Agilent Feature Extraction software (version 11.0.1.1). Limma package for R software normalized and processed the expression data. Differences in the fold change, *P*‐value, and raw intensity were used to identify significant differentially expressed circRNAs between the two groups.

# Quantitative Real-Time PCR

# Exosomal RNAs were extracted using the Trizol LS reagent (Invitrogen, USA) as per the manufacturer’s protocol, and subsequently reverse-transcribed into complementary DNA (cDNA) with a PrimeScript RT Reagent Kit (Bio-Rad, USA). The cDNA was detected on a CFX connect Real-Time PCR system using the SYBR Green Real-time PCR Master Mix Kit (Bio-Rad, USA). All assays were conducted in 3 separate RTs, and the transcript levels were normalized to the expression of β-Actin. Specific primers for qPCR are as follows:

# hsa_circ_0021647-F: 5'-CAATCCATGCAAACGGTGGT-3'

# hsa_circ_0021647-R: 5'-GCCATAAGGTCTTGTACTCG-3'

# hsa_circ_0000288-F: 5'-GCGTCTCAGCCTCAAGTATT-3'

# hsa_circ_0000288-R: 5'-GGATGATACCAAAGGAACCGT-3'

hsa_circ_0000367-F: 5'-CATTCCCTGCACTCATCGAA-3'

# hsa_circ_0000367-R: 5'-GAACAGAGTGCTTACTGGGAC-3'

# β-actin-F：5'-AGAGCTACGAGCTGCCTGAC -3'

# β-actin-R：5'-AGCACTGTGTTGGCGTACAG -3'

**RNase R treatment**

A total of 2.5 μg extracted RNAs were treated with 2 U/μg of RNase R (Geneseed, China) at 37°C for 10 min, followed by incubation at 70°C for 10 min to inactivate RNase R. After reverse transcription, circRNAs and their linear counterparts were characterized using quantitative PCR kit (Bio-Rad, USA).

**Cell culture and transfection**

The human CCA cell lines (CCLP1 and Huh28) were kindly provided by Professor Yunfu Cui (Second Affiliated Hospital of Harbin Medical University), and cultured in Dulbecco’s modified Eagle’s medium (Hyclone, USA) containing 10% fetal bovine serum (Hyclone), 25 U /ml penicillin (Hyclone), and 25g/ ml streptomycin (Hyclone). All cells were tested as mycoplasma-negative by PCR before use at Research Center for Biliary Diseases of West China Hospital.

The CCA cells were transfected with [antisense oligonucleotide](javascript:;) (ASO) or negative control (Ribo-Bio, China) using Lipofectamine 3000 reagent (Invitrogen, USA) as per the manufacturer’s protocol. The sequences of ASO are as follows:

ASO-h-hsa-circ-0021647: TGCAAACGGTGGTAAATTCA

ASO-h-hsa-circ-0000367: GACTTGTCCAGGTCCATTCC

ASO-h-hsa-circ-0000288: TCCATGCAAACGGTTCCTTT

**Cell viability assay**

The cell viablity was examined using the Cell Counting Kit-8 (CCK8, Biosharp, China). CCA cells were seeded into 96-well plates at a density of 5 × 10^3^ cells/ well. Transfection of ASO was performed thereafter. After 24, 48, 72, and 96 hours of culture, CCK-8 reagent (10 μL per 100 μL of the FBS-freed medium) was added into each well as per the kit protocol and incubated for 1 hour at 37°C. The absorbance was measured with a microplate reader (BioTek, USA) at an optical density (OD) of 450 nm.

**Transwell assay**

Using a Transwell chamber, 500 μL medium + 10% FBS was inserted into the bottom chamber, and the CCA cells transfected in 200 μL serum-free medium were placed in the upper chamber (Corning, USA) at a density of 10^5^ cells/well. After incubating the cells at 37°C for 48 h, they were fixed within the membrane with 4% paraformaldehyde followed by crystal violet staining. Any cells that had migrated were imaged and quantified. Finally, the images were visualized under a microscope. The invasion assay method followed the same procedure, except that 50 μL Matrigel (BD Biosciences, USA) was used to coat the upper chamber.

**EdU assay**

A Cell-Light EdU DNA Cell Proliferation Kit (RiboBio, China) was used to assess cellular proliferation. CCA cells were incubated overnight at a density of 2 × 10^4^ cells/well in 96-well plates, followed by transfection with ASO. After the cells had undergone a 2 h incubation in EdU for 2 h, they were fixed in 4% neutral paraformaldehyde. Apollo dye solution (Beyotime, China) was used to stain the cells and Hoechst 33342 (Thermo, USA) was used to stain for nucleic acids. Evaluation of cellular EdU incorporation was performed with a fluorescence microscope (Olympus, Japan) followed by analysis with Image-Pro Plus software.

**Annexin V/PI apoptosis assay**

Cells were seeded into 6-well plates at a density of 1 × 10^6^ cells/well. Following the designated ASO treatments, all cells including both floating and attached cells were collected by trypsinization (Gibco 0.25% Trypsin, without EDTA) and washed with PBS. The apoptotic cells were detected by Annexin V-FITC Apoptosis Detection Kit (4A biotech Co. Ltd., China) by staining with Annexin V-FITC and PI according to the supplier’ s instructions. Viable and dead cells were detected by a CytoFLEX flow cytometer (CytoFLEX Flow Cytometer, Beckman Coulter, Brea, CA, USA).

**Tumor growth assay in vivo**

Male BALB/c nude mice (aged 4-6 weeks, weighted 18-20 g) were purchased from Dossy Experimental Animals Corporation (Chengdu, China), and maintained under SPF conditions at the experimental animal center of West China Hospiatal of Sichuan University. Mice were subcutaneously injected with CCLP-1 cells (1×${10}^{6}$ cells in 100 μL of sterile PBS) in the right flanks. One week after injection, the tumor size reached approcimately 100 mm3, and mice were randomly divided into 4 groups (n=5 in each group). ASOs were delivered according to groups by intratumor injection at a dose of 10 nmol (100 μL, 100 mM ASO in 100 μL of sterile PBS) each mouse every 3 days. Tumor volume was measured by caliper every 3 days and calculated as (length×$\mathrm{width}^{2}$)/2. At the end of the experiment, mice were sacrificed, and subcutaneous tumors were harvested. Proteins were extracted for immunoblotting analysis, and consecutive sections were made to perform immunohistochemistry staining.

All animal experiments were performed in accordance with a protocol approved by our Institutional Animal Care and Use Committee (IACUC). The randomization of animal allocation was done by random numbers generated by the computer. Following experimentation, no animals were excluded from analysis, and no blinding procedure was undertaken. The reporting of mouse studies in this manuscript conforms with the Animal Research: Reporting of In Vivo Experiments (ARRIVE) guidelines.

**Immunochemistry (IHC)**

Harvested CCLP-1 xenograft tissues of BALB/c nude mice were collected, fixed in formalin and embedded in paraffin to obtain formalin-fixed, paraffin-embedded (FFPE) tissue samples, and 4-μm-thick sections were then prepared for IHC. All slides were dewaxed and dehydrated, and the endogenous peroxidase activity was then quenched with 3% hydrogen peroxide for 10 min. Antigen retrieval was achieved by covering the slides with citrate buffer (pH 6.0) and heating for 10 min at 95 °C. The sections were then incubated with 10% goat serum albumin for 2 h at room temperature to block nonspecific binding and then incubated with Ki-67 (WanleiBio, 1:100), Vimentin (WanleiBio, 1:200), β-catenin (WanleiBio, 1:200) and E-cadherin (WanleiBio, 1:200) antibodies. After overnight incubation at 4 °C and three washes with PBS, the sections were incubated with the secondary antibody for 1 h at room temperature and rinsed in PBS. Diaminobenzidine (DAB) was used as a chromogen, and the sections were counterstained with hematoxylin. The stained samples were examined by a Nikon Eclipse Ti2 microscope (Nikon Instruments, Melville, NY, USA).

**Construction of circRNA–miRNA–mRNA regulatory network**

CircRNA-miRNA interaction was predicted using circAtlas.^2^ The overlapping miRNAs between each target circRNA was analyzed in Diana tools to identify miRNA-mRNA interactions.^3^ The overlapping genes between the predicted miRNA target genes and the DEGs were obtained for circRNA–miRNA–mRNA network construction. The Cytoscape 3.10.1 software was used to visualize the regulatory network.

**Surgical procedures and systemic therapy**

Patients who were evaluated as surgical candidates underwent curative-intent resection of CCA. Radical resection for perihilar cholangiocarcinoma was applied for pCCA and iCCA involving the hepatic hilus, while the Whipple procedure was applied for dCCA. Please note that the type of resection and indication for surgery were not related to the following method adopted:

In short, patients were placed in supine position, followed by total intravenous general anesthesia. Laparotomy was performed through a reverse L-incision. Cavitron ultrasonic surgical aspirator or clamp crushing was used as the main methods for parenchyma transections, Pringle maneuver and bipolar electrocoagulation were usually applied to control blood loss. For vessels ≥5 mm in diameter, Hem-o-lock clips (Weck Surgical Instruments, USA) or Titanium clips were used to achieve vascular control. The hepatic veins and portal pedicles were transected by a laparoscopic linear stapler. Abdominal lymph nodes were routinely dissected for biopsy. The resected specimens were then placed into a protective bag and extracted through an enlarged port in the upper abdomen or the suprapubic transverse incision. Abdominal drainage was routinely placed on the cut surface.

After surgical procedure, biopsy was performed to reveal pathological details of the tumor and the dissected lymph nodes. Margin distance, presence of microvascular invasion, macrovascular invasion, and lymph node metastasis were evaluated according to previously published standards. Macrovascular invasion (MaVI) is defined as tumor invasion into a major vessel that can be identified by radiological imaging or macroscopic examination.^4^ The diagnosis of microvascular invasion (MiVI) is accessed through histological examination.^5^ Tumor histological grade and TNM staging was assessed according to AJCC 8^th^ grading system.^6^ A double-drug regimen of gemcitabine plus cisplatin was routinely applied postoperatively.

**Postoperative follow-up**

After discharge, all patients were followed every 1-3 months in the first year and every 3 months thereafter. Routine blood tests, liver function tests, tumor marker tests as well as abdominal ultrasonography were routinely measured in follow-ups. CT or MRI was performed as soon as the recurrence was suspected in ultrasound imaging. Telephone interviews were conducted in order to make sure the health condition of patients. All data was de-identified before admitted into the database.

The primary endpoint of this study was recurrence-free survival (RFS), while the secondary endpoint included overall survival (OS) and complication incidence. RFS was defined as the time from curative-intent surgical resection to either death or CCA recurrence; OS was defined as the time from curative-intent surgical resection to death.

**Statistical analysis**

In this study, qPCR expression levels of circRNAs were calculated by absolute quantification and presented by median with range. Data normality was verified by Shapiro-Wilk test. Cut-off value was determined by optimal Youden’s index. Combined diagnostic model was constructed via logistic regression analysis (stepwise optimization approach used for model optimization) and evaluated by receiver operating characteristic (ROC) curves, sensitivity, specificity and accuracy index (AI). DeLong test was used to compare statistical difference between ROC curves. Spearman correlation matrix was used to confirm the independency between different biomarkers with nonnormal distribution. Survival analysis was accessed through the Kaplan-Meier method and comparison between groups was based on the log-rank test. Cox PH model was applied to establish the prognostic models (COX PH assumption verified at first). Continuous variables with normal distribution were presented as mean value with standard deviation, while other continuous variables were presented as median with range. Categorical variables were presented as frequencies along with percentages. Pearson's chi-squared test (χ2) was conducted to analyze categorical variables while independent samples t or Mann-Whitney U test was used for continuous variables. Random forest model was used to manage missing data in CA19-9 (8 out of 389 records, 2%) to complete multiple linear regression. The “mice” package in R was applied for missing data management. All tests were two-sided, and a p value of less than 0.05 was defined as statistically significant

Data analysis was performed with R 4.1.3 (R Foundation for Statistical Computing, Vienna, Austria) using “rstatix”, “glm”, “rms”, “ggplot2”, “survminer” and other functions/packages. Data analysis and visualization concerning box plots, heatmap and ROC curves were performed using GraphPad prism software (v7, GraphPad software, USA).

**Supplementary Figures and Tables**

**
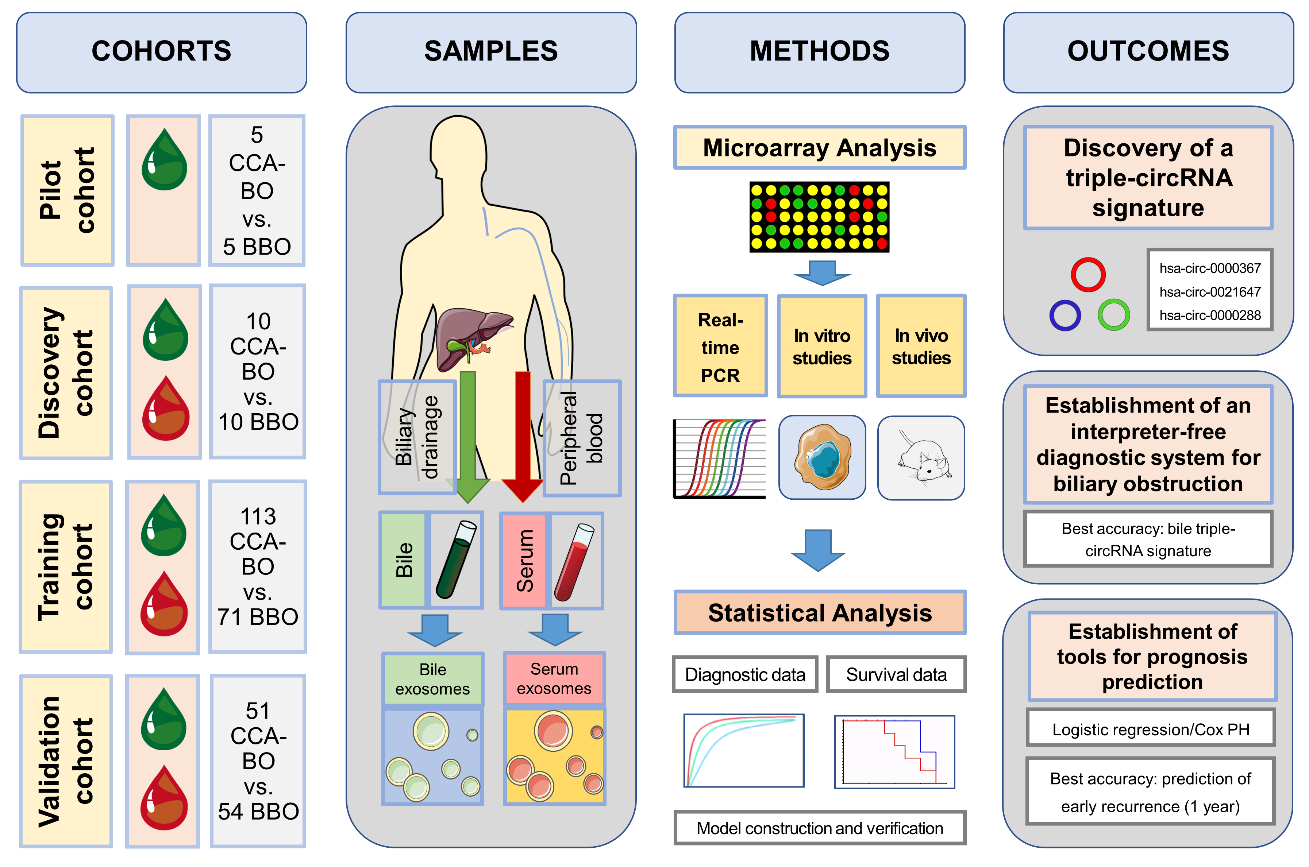
**

**Supplementary Fig. 1 Graphical abstract of this study.**


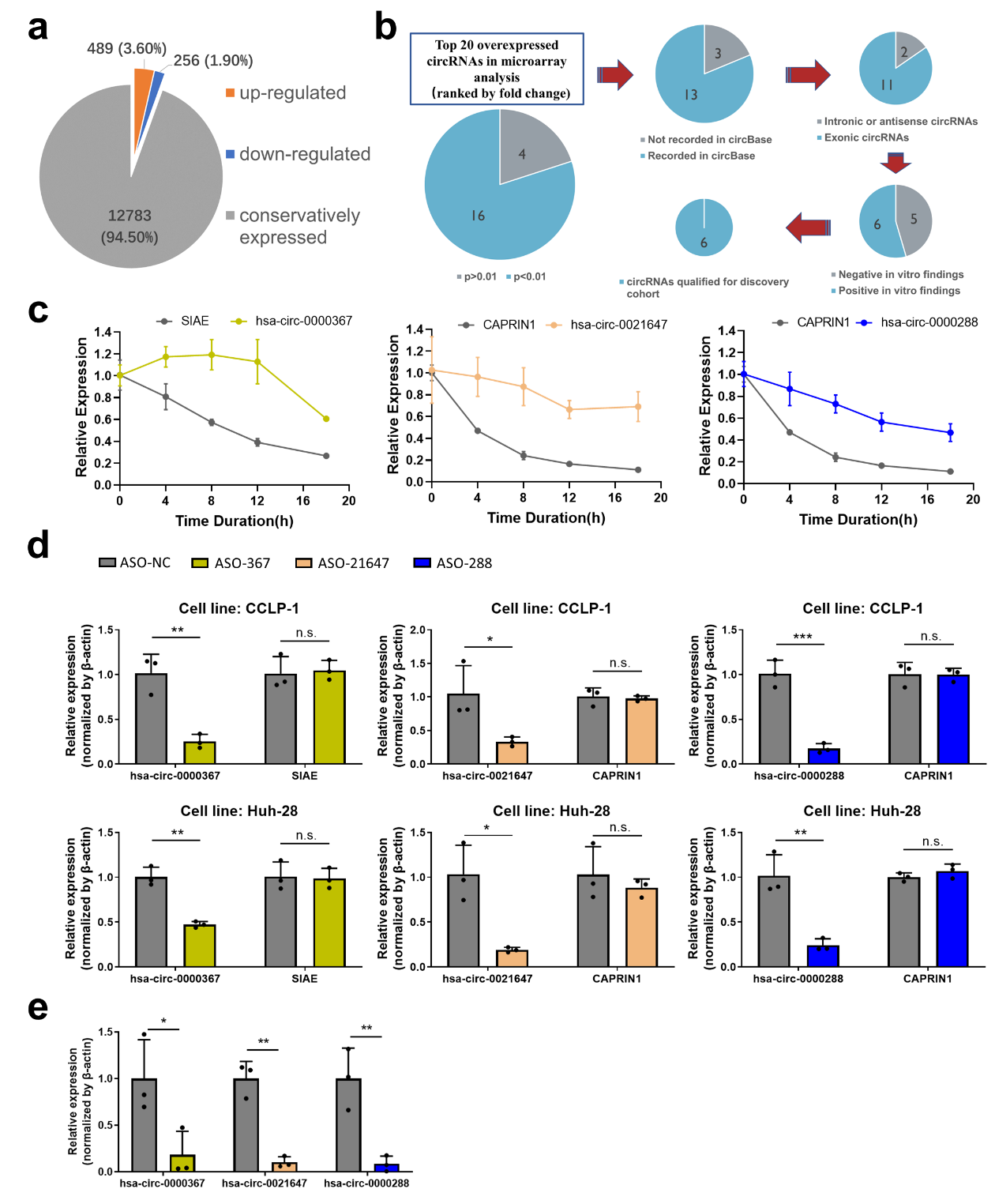


**Supplementary Fig. 2 Screening of candidate circRNAs and verification of ASO knockdown.** **(a)** Summary of differentially enriched circRNAs in CCA-BO compared with BBO. **(b)** Summary of the screening process of 6 candidate circRNAs. **(c)** Dactinomycin treatment of qPCR products verified the circularity of target circRNAs. **(d)** ASO successfully knocked down target circRNAs without affecting their host genes. **(e)** ASO treatment successfully knocked down target circRNAs *in vivo*.


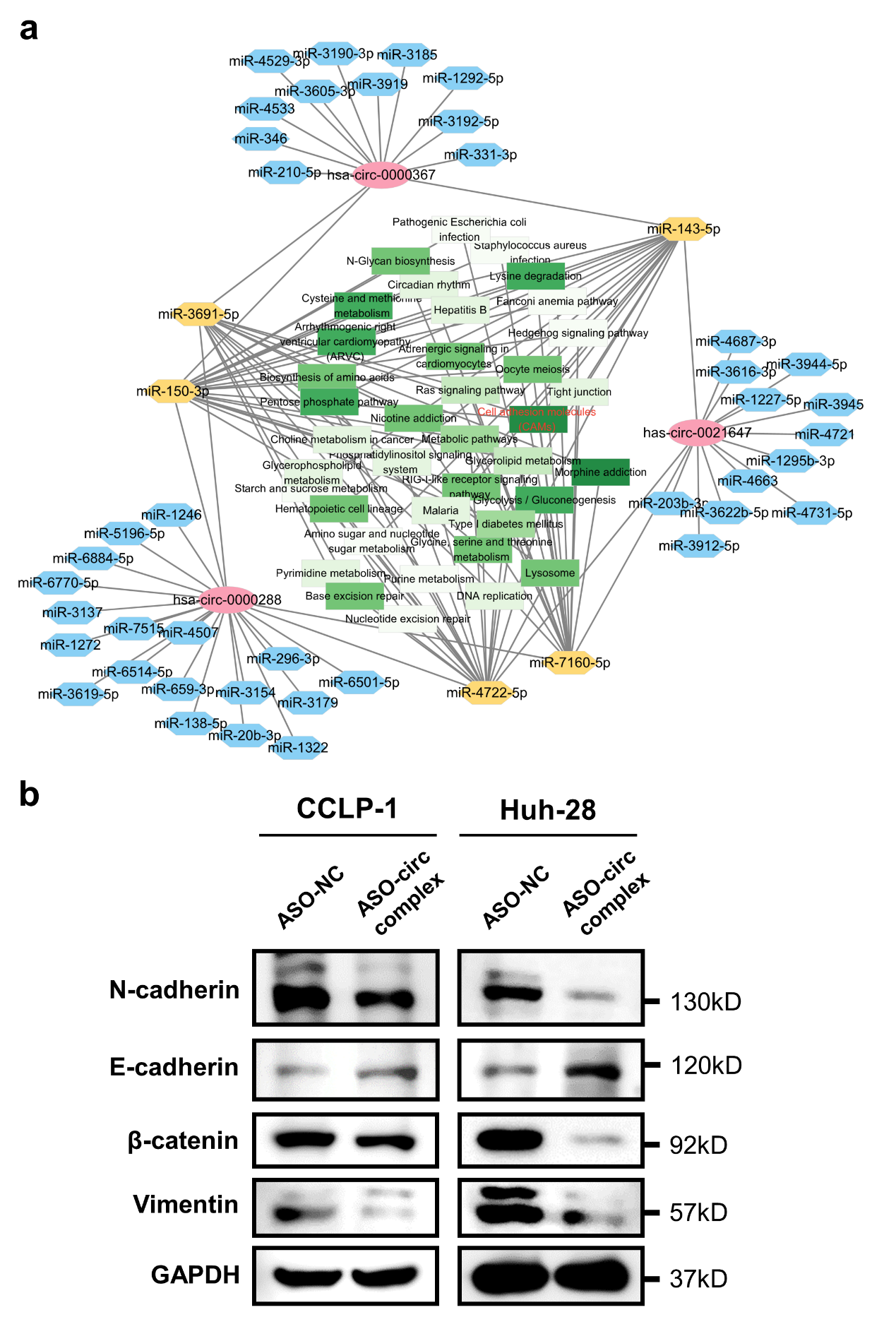


**Supplementary Fig. 3 Preliminary exploration of downstream functions of target RNA.** **(a)** A circRNA–miRNA–mRNA network using circAtlas and Diana-miRPath. P value was used for the ranking of miRNA enrichment pathways. **(b)** Treatment of circRNA-ASO complex led to down-regulation of N-cadherin and regulation of other EMT markers in two independent CCA cell lines.


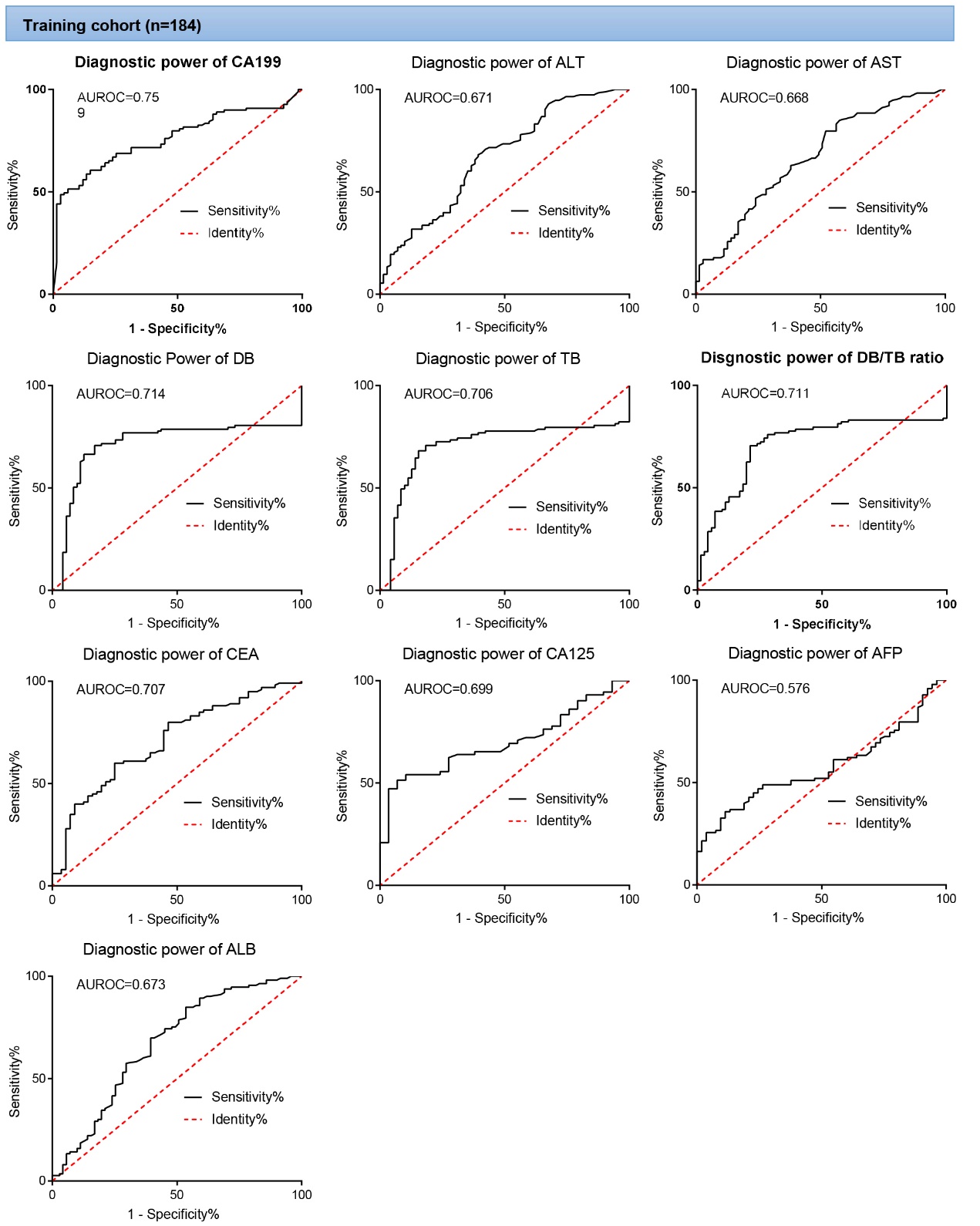


**Supplementary Fig. 4 Diagnostic power of currently used clinical indices in CCA-BO diagnosis.** ROC curves of currently used clinical indices for the diagnosis of CCA-BO in the training cohort. CA19-9 was evaluated as the best biomarker for CCA-BO diagnosis but still far from satisfactory (AUROC=0.759).


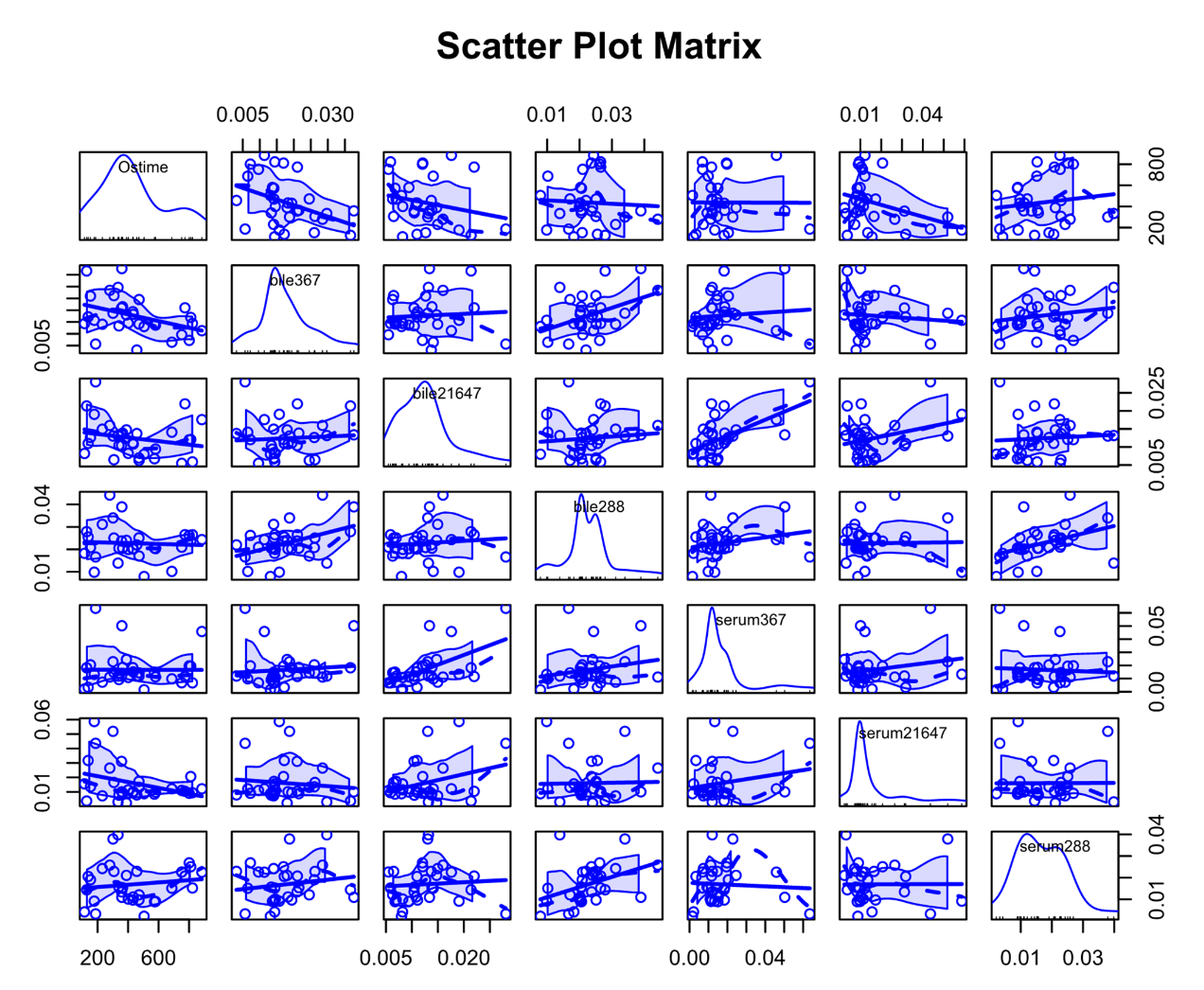


**Supplementary Fig. 5 Scatter plot matrix revealed a moderate correlation between the abundance of each circRNA in bile exosomes and its serum counterparts.**


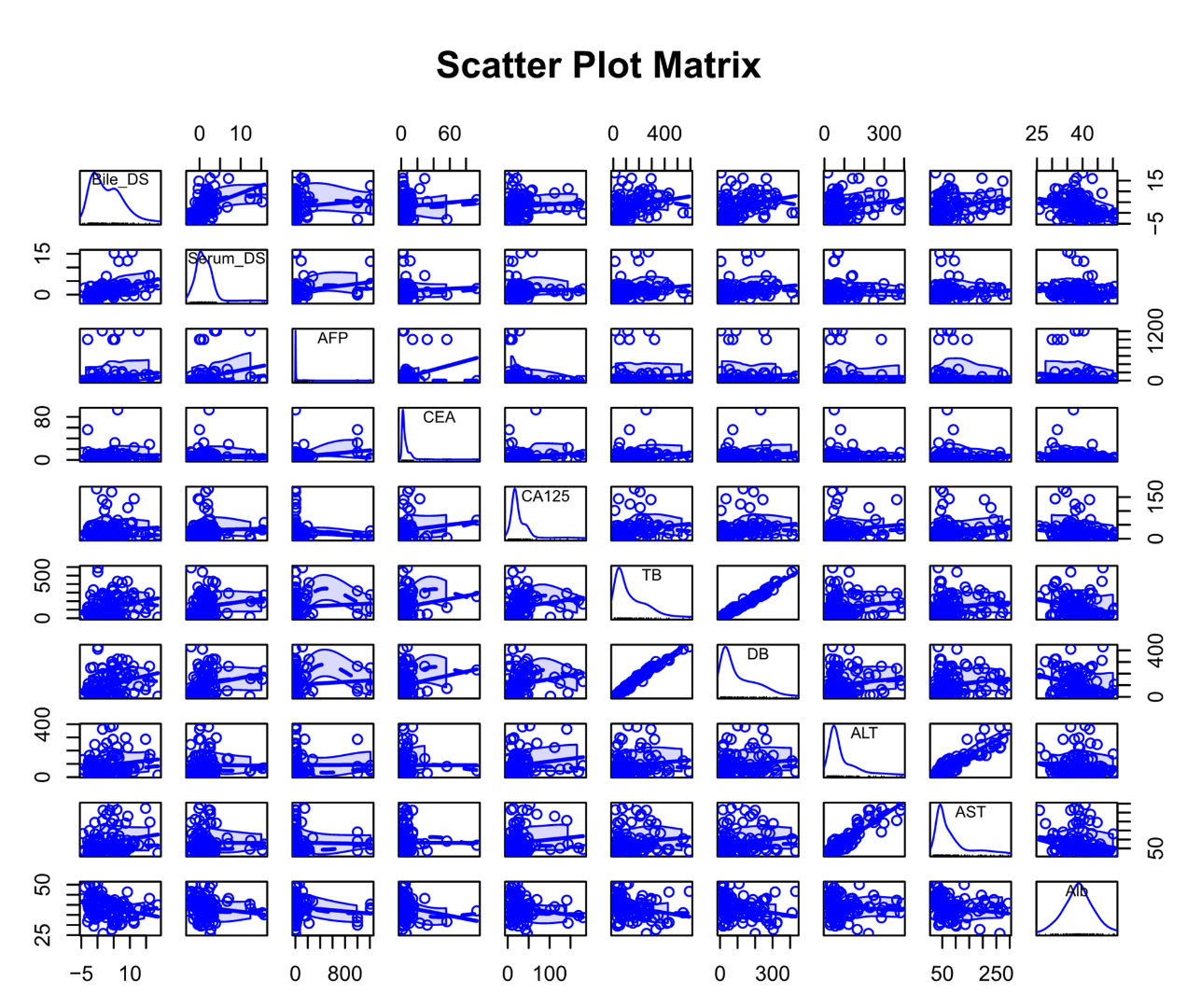


**Supplementary Fig. 6 Scatter plot matrix revealed a strong correlation between Bile-DS and Serum DS while independent from other factors.**


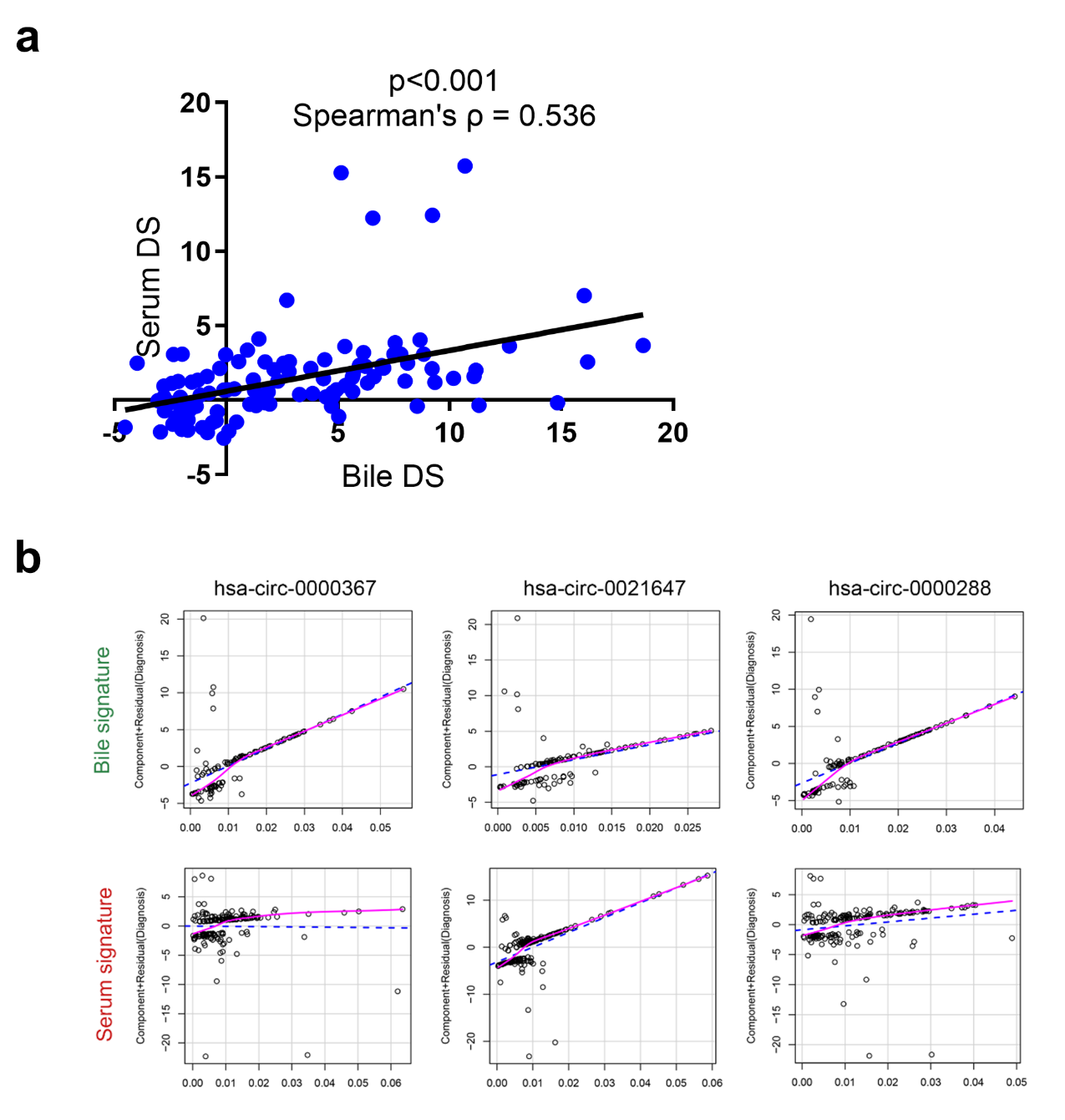


**Supplementary Fig. 7 Goodness-of-fit evaluation of the circRNA-based diagnostic models.** **(a)** There was a strong to moderate correlation between Bile-DS and Serum-DS (Spearman’s ρ= 0.536). No significant correlation was found between the diagnostic scores and other clinical indices. **(b)** Component plus residual plots of bile and serum diagnostic models.


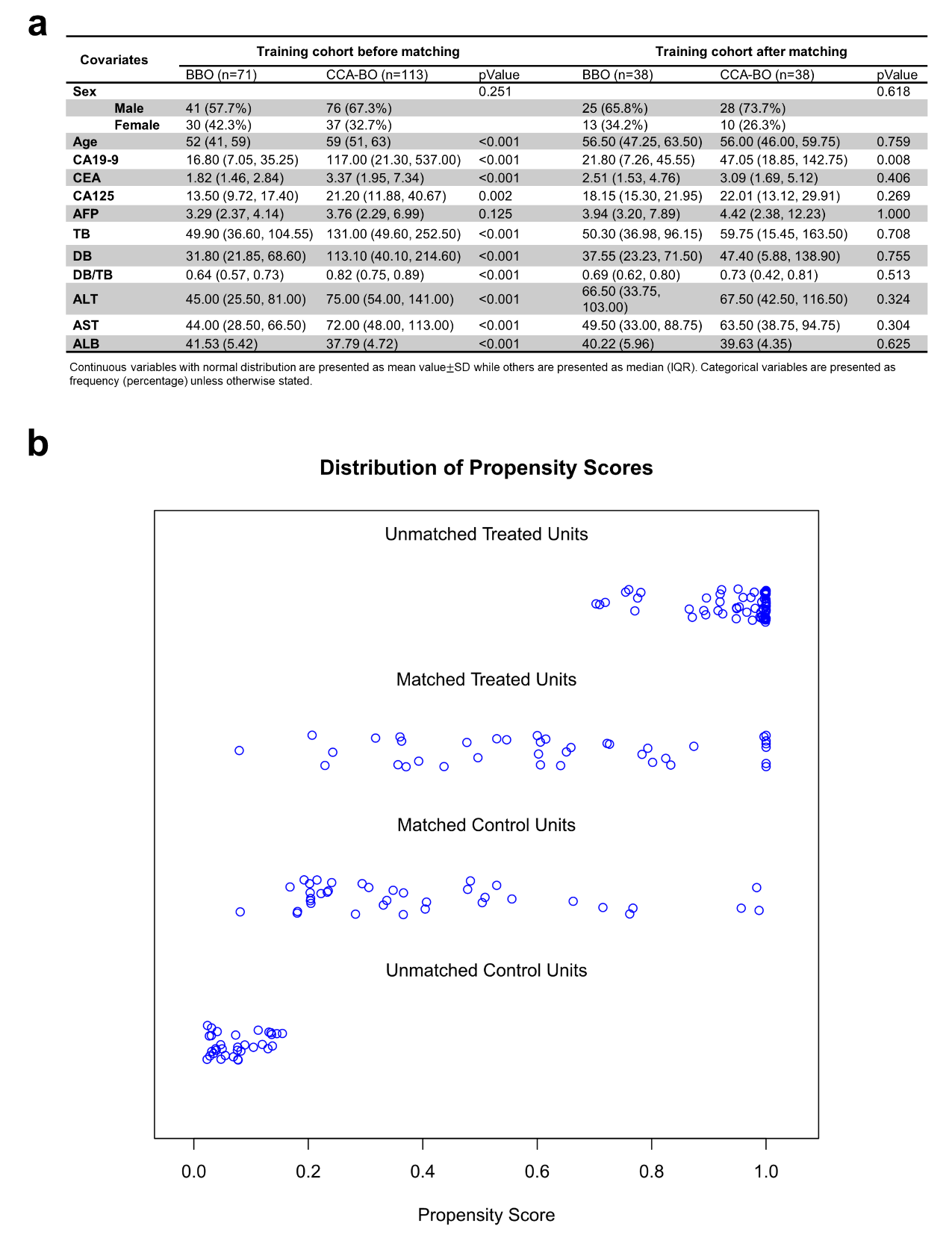


**Supplementary Fig. 8 CircRNA-based diagnostic models remained powerful after propensity score matching in the training cohort.** A propensity score match (PSM) analysis was performed in the training cohort to simulate more difficult diagnostic scenarios. **(a)** Baseline characteristics following PSM showed comparable baseline between BBO and CCA-BO patients except for CA19-9. **(b)** The matched training cohort.


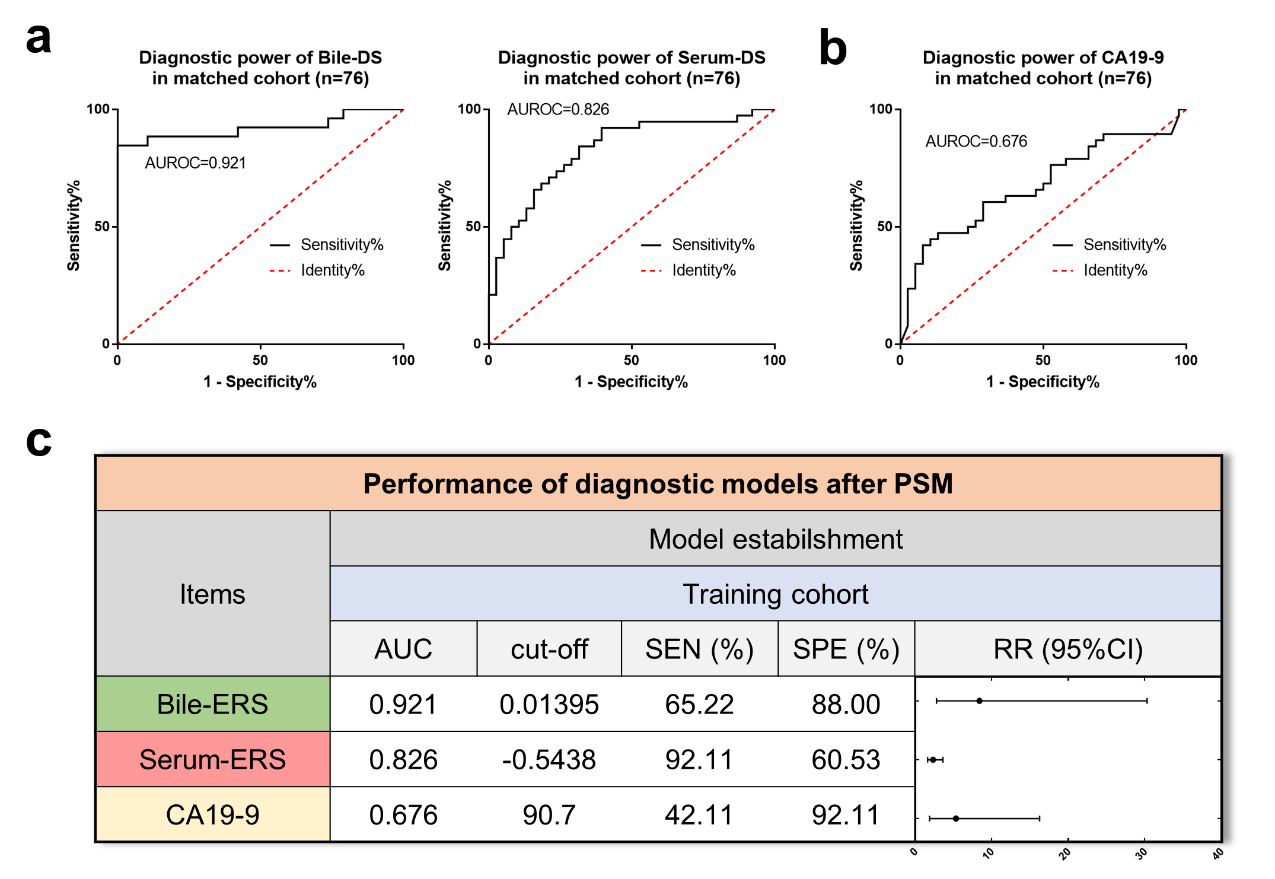


**Supplementary Fig. 9 CircRNA-based diagnostic models remained powerful after propensity score matching in the training cohort.** In the matched training cohort, diagnostic power of Bile-DS and Serum-DS remained robust **(a)**, while **(b)** CA19-9 showed poor diagnostic power. **(c)** Summary of diagnostic performance following PSM in the training cohort.


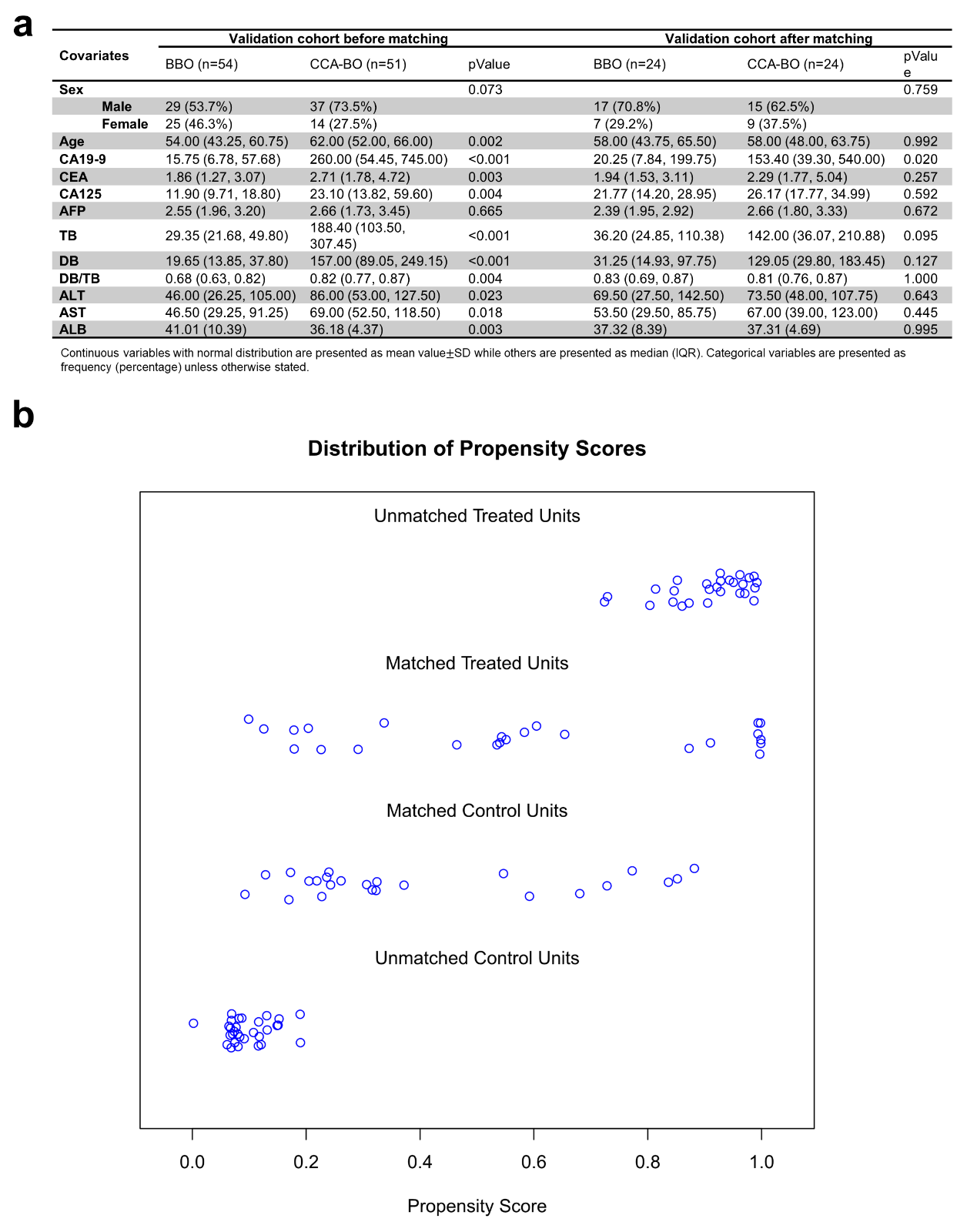


**Supplementary Fig. 10 CircRNA-based diagnostic models remained powerful after propensity score matching in the validation cohort.** Similarly, a propensity score match (PSM) analysis was performed in the validation cohort. **(a)** Baseline characteristics following PSM showed comparable baseline between BBO and CCA-BO patients except for CA19-9. **(b)** The matched validation cohort.


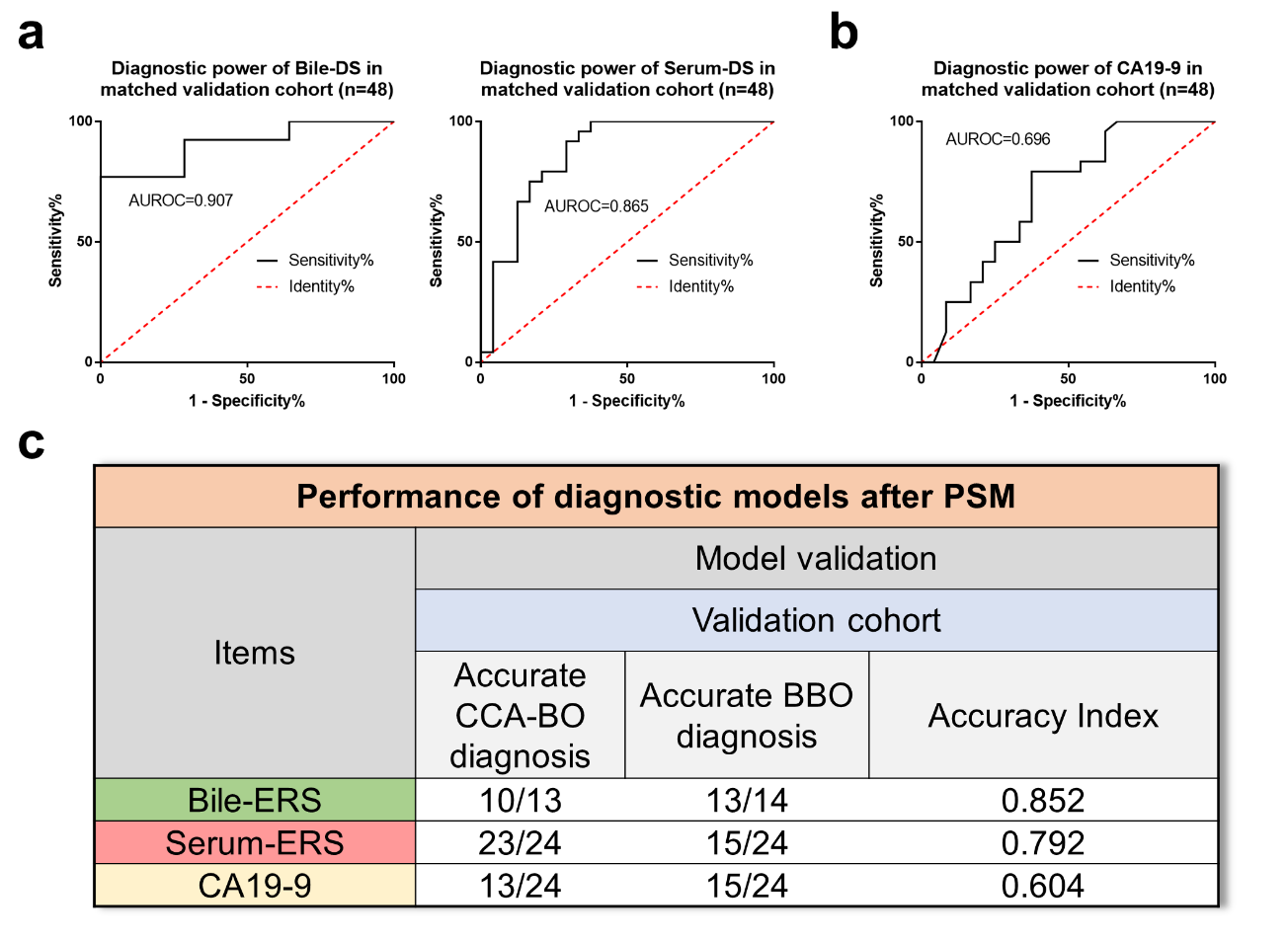


**Supplementary Fig. 11 CircRNA-based diagnostic models remained powerful after propensity score matching in the validation cohort.** In the matched validation cohort, diagnostic power of Bile-DS and Serum-DS remained robust **(c)**, while **(d)** CA19-9 showed poor diagnostic power in the matched cohort. **(e)** Summary of diagnostic performance following PSM in the validation cohort.


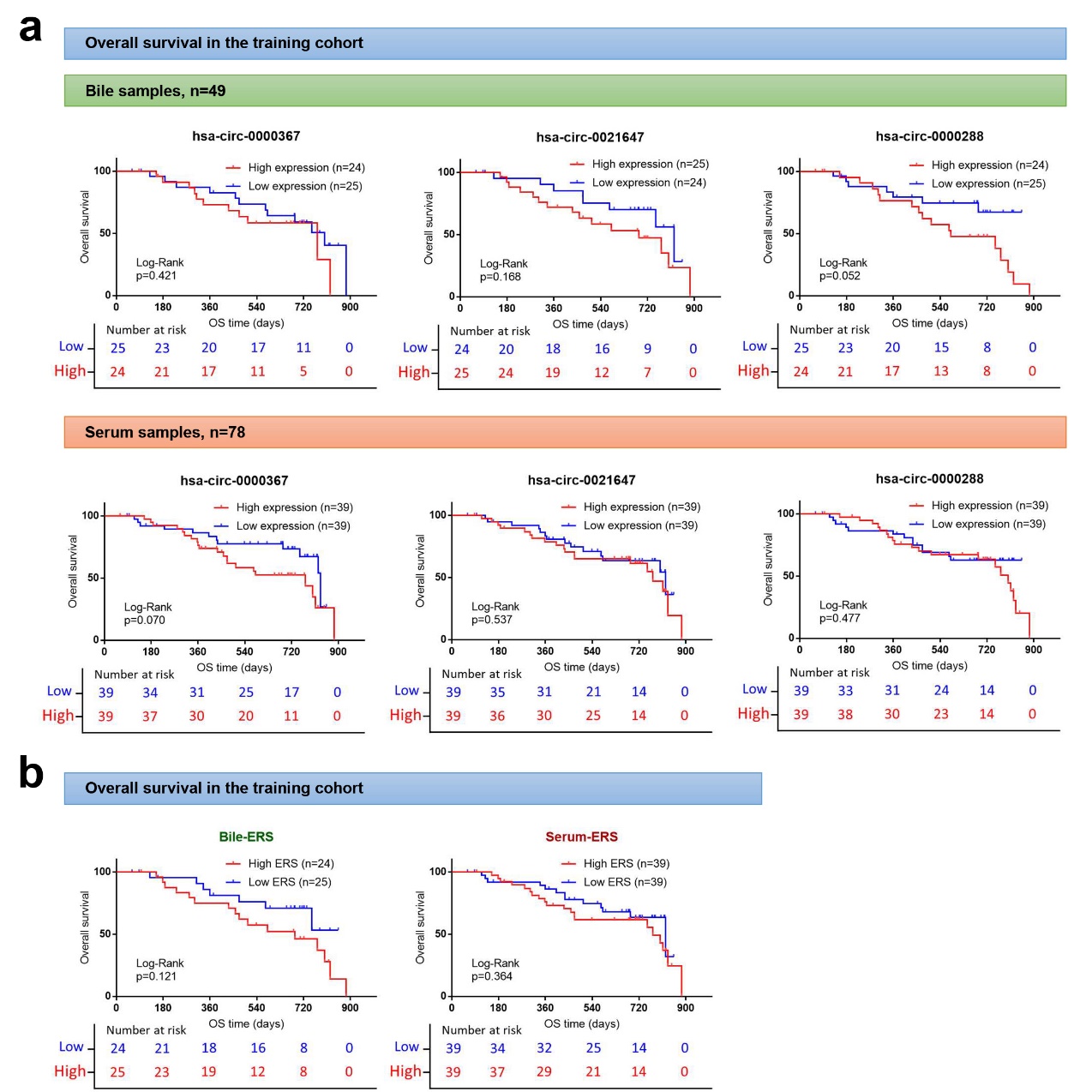


**Supplementary Fig. 12 Overall survival of CCA-BO patients undergoing curative-intent treatment in the training cohort.** **(a)** CCA-BO patients were grouped by the expression level of each target circRNA and compared of overall survival (OS). **(b)** CCA-BO patients were grouped by the Bile-ERS and Serum- ERS levels and compared of OS.


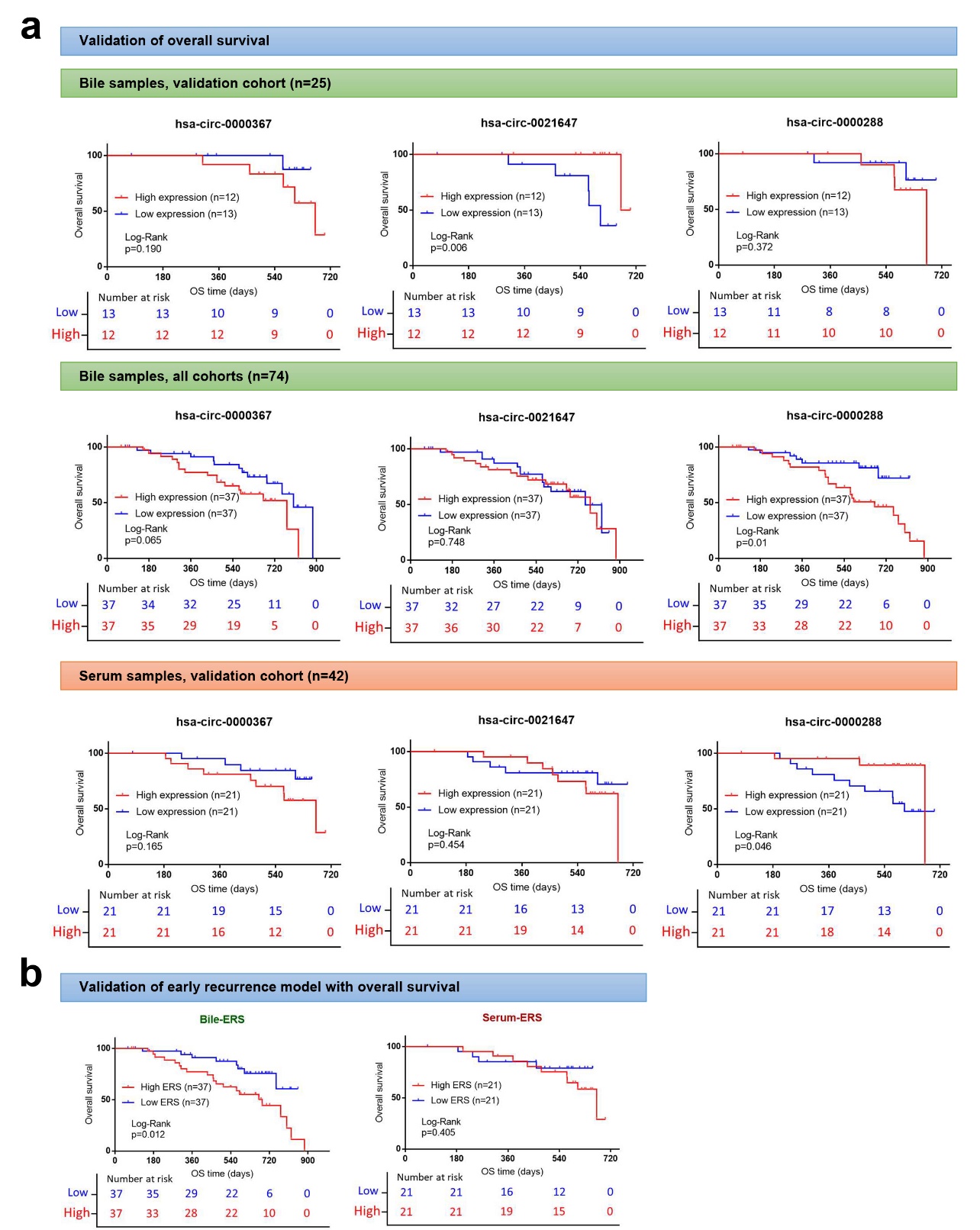


**Supplementary Fig. 13 Overall survival of CCA-BO patients undergoing curative-intent treatment in the validation cohort.** **(a)** Among the three target circRNAs, only hsa-circ0000288 was potentially related to OS of CCA-BO patients. Bile samples in the validation cohort were insufficient for independent verification and were used as expansions of the training cohort. **(b)** Bile-ERS potentially correlates to OS of CCA-BO patients. To sum up, Fig. S6 and Fig. S7 shows that the relationship between the expression levels of target circRNAs and OS of CCA-BO patients was indistinctive.


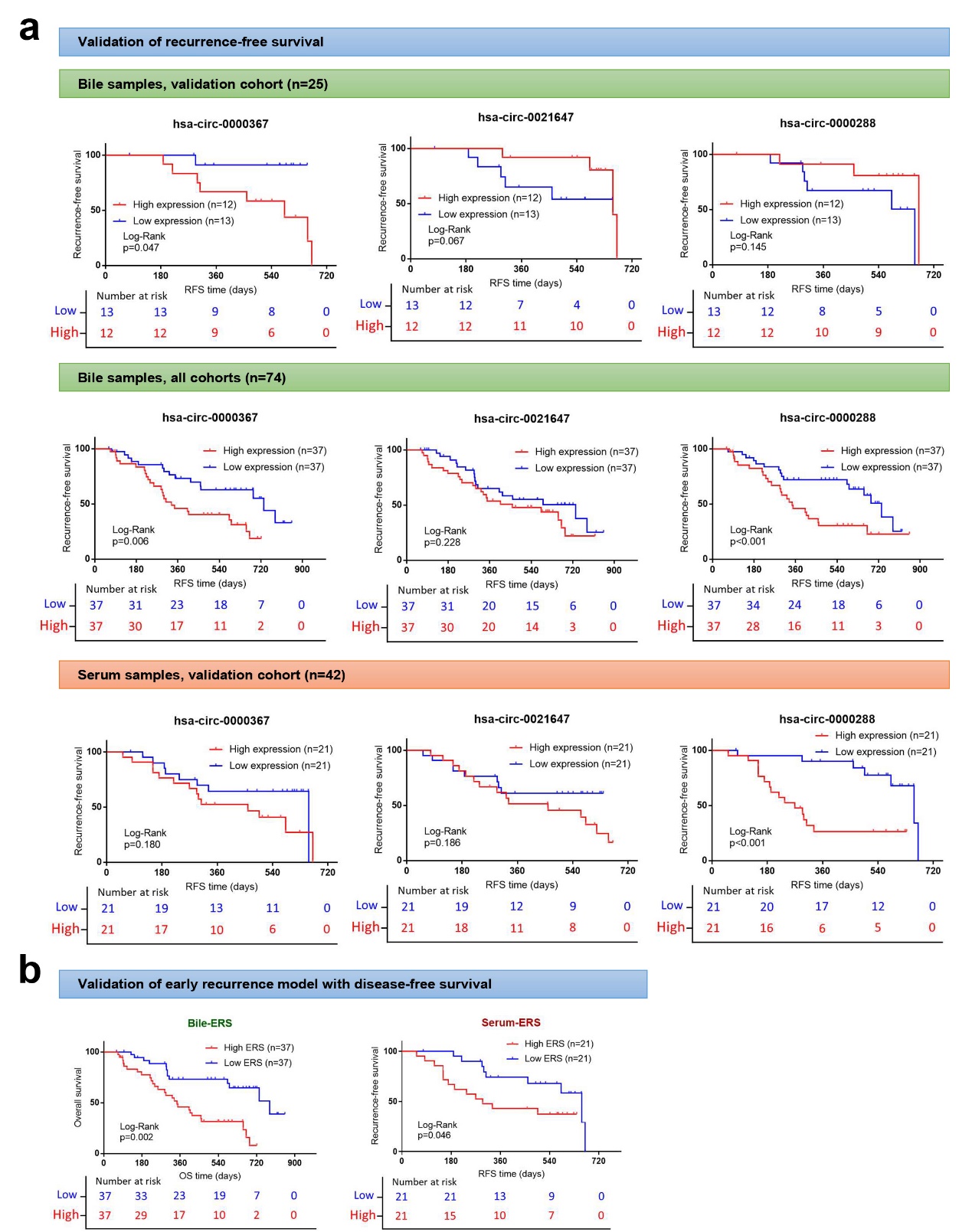


**Supplementary Fig. 14 Recurrence-free survival of CCA-BO patients undergoing curative-intent treatment in the validation cohort.** **(a)** Expression levels of individual circRNAs showed better correlation to RFS compared with OS, verified by the validation cohort. **(b)** Bile-ERS strongly correlates to RFS of CCA-BO patients. Serum-ERS is also predictive of RFS. To sum up, the early recurrence model based on target circRNAs is predictive of CCA recurrence following curative-intent surgery


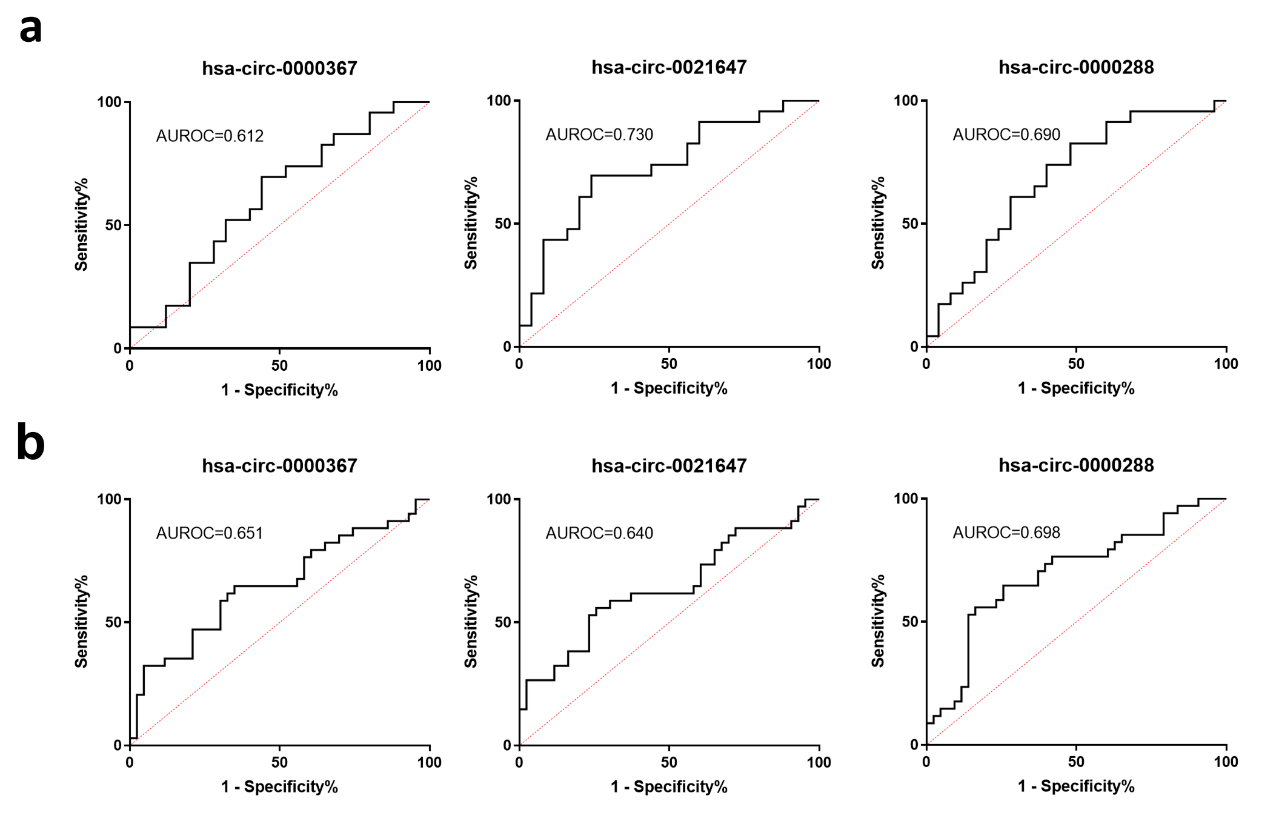


**Supplementary Fig. 15 Predictive effect of individual circRNAs.** Predictive effect of individual circRNAs on early recurrence was unremarkable regarding both bile **(a)** and serum **(b)** exosomes.


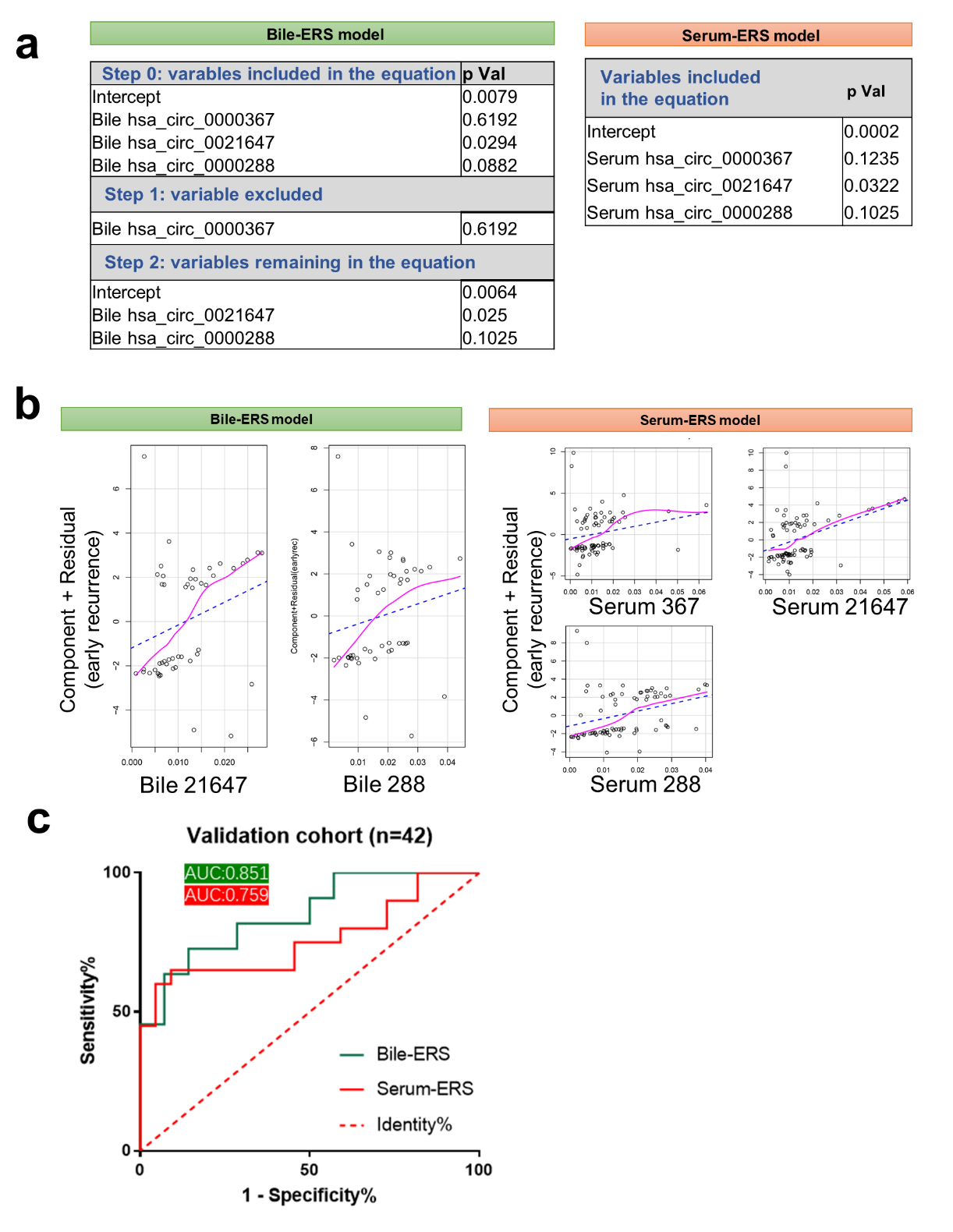


**Supplementary Fig. 16 Establishment of early recurrence models.** **(a)** Stepwise logistic regression excluded bile exosomal hsa-circ-0000367 to setup a formula for ERS calculation, its goodness-of-fit was verified by **(b)** component plus residual plots. **(c)** Bile-ERS and Serum-ERS performed well predicting early recurrence in the validation cohort, evaluated by AUROC.


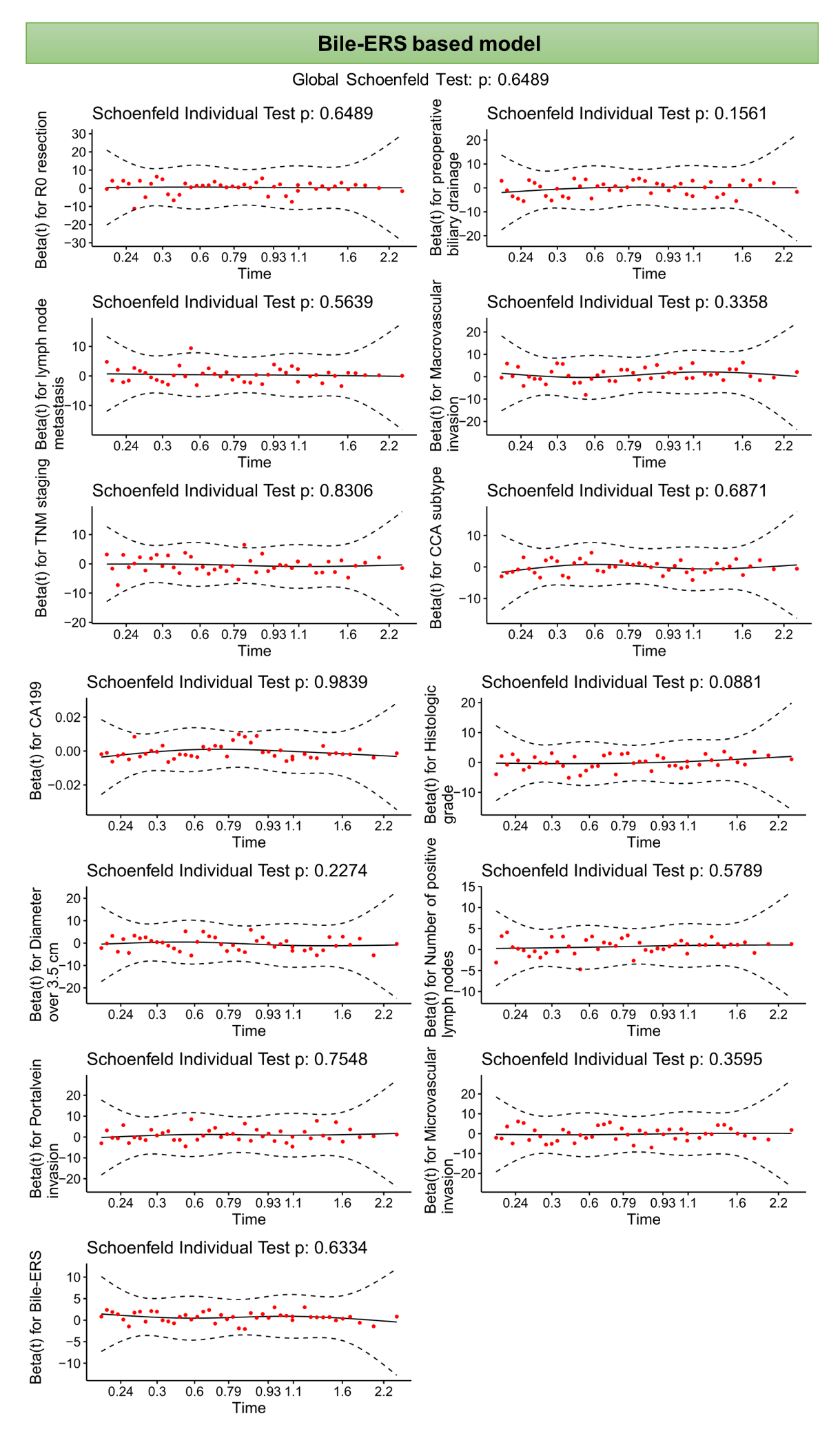


**Supplementary Fig. 17 Schoenfeld's global test to evaluate the proportional hazards assumption for Bile-ERS based model.**


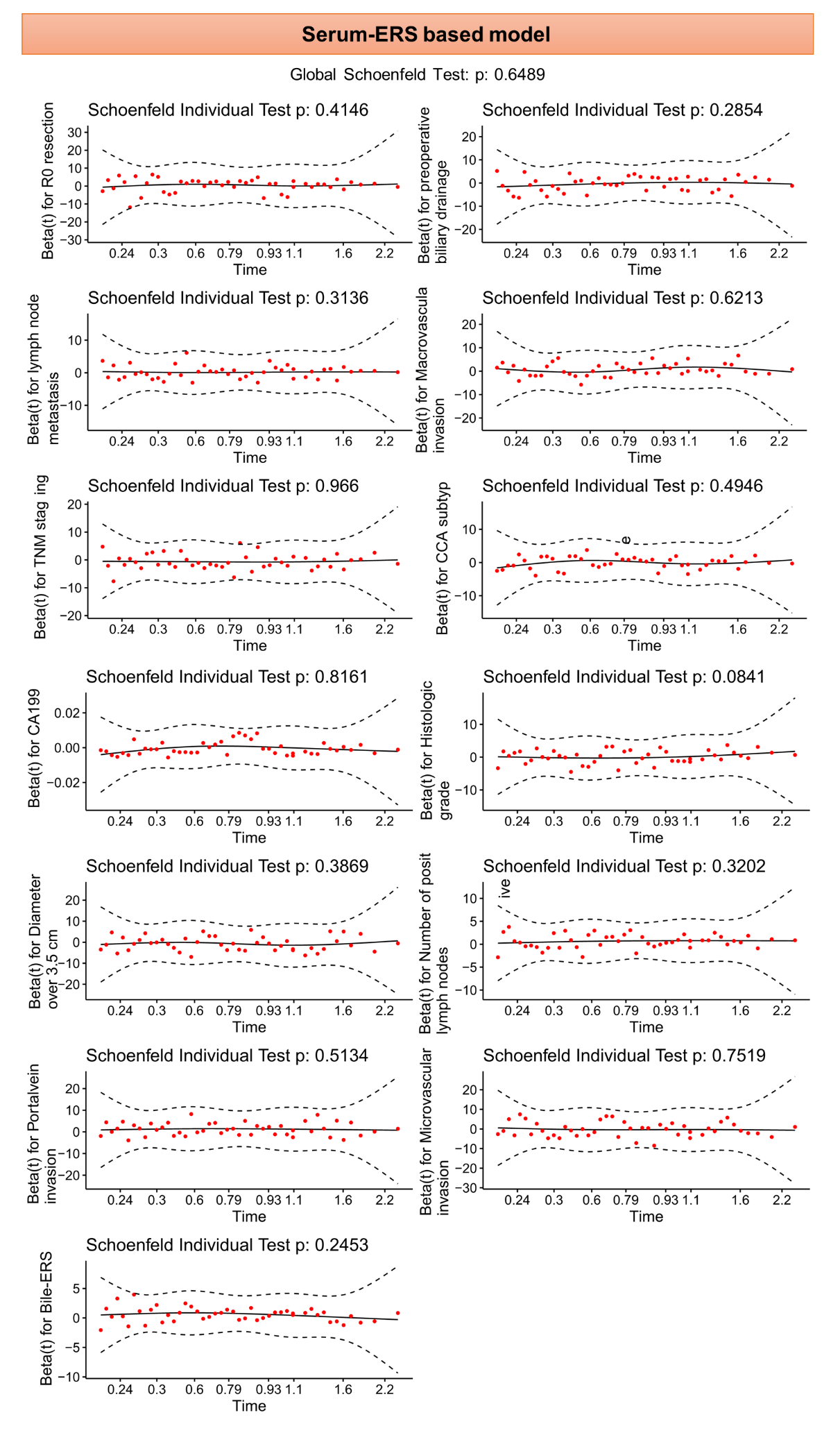


**Supplementary Fig. 18 Schoenfeld's global test to evaluate the proportional hazards assumption for Serum-ERS based model.**


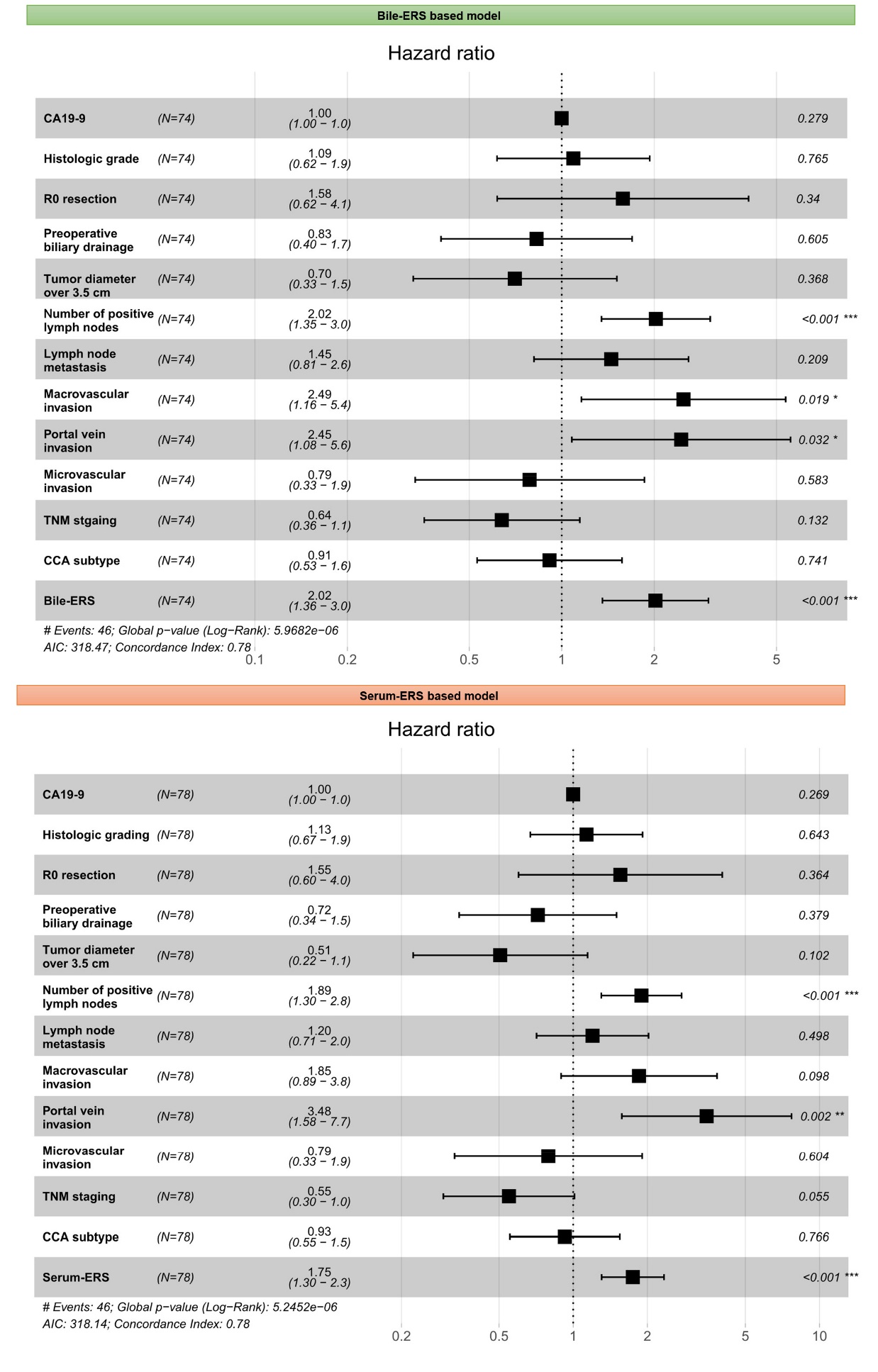


**Supplementary Fig. 19 Forest plots of Cox proportional hazards models based on Bile-ERS and Serum-ERS.**


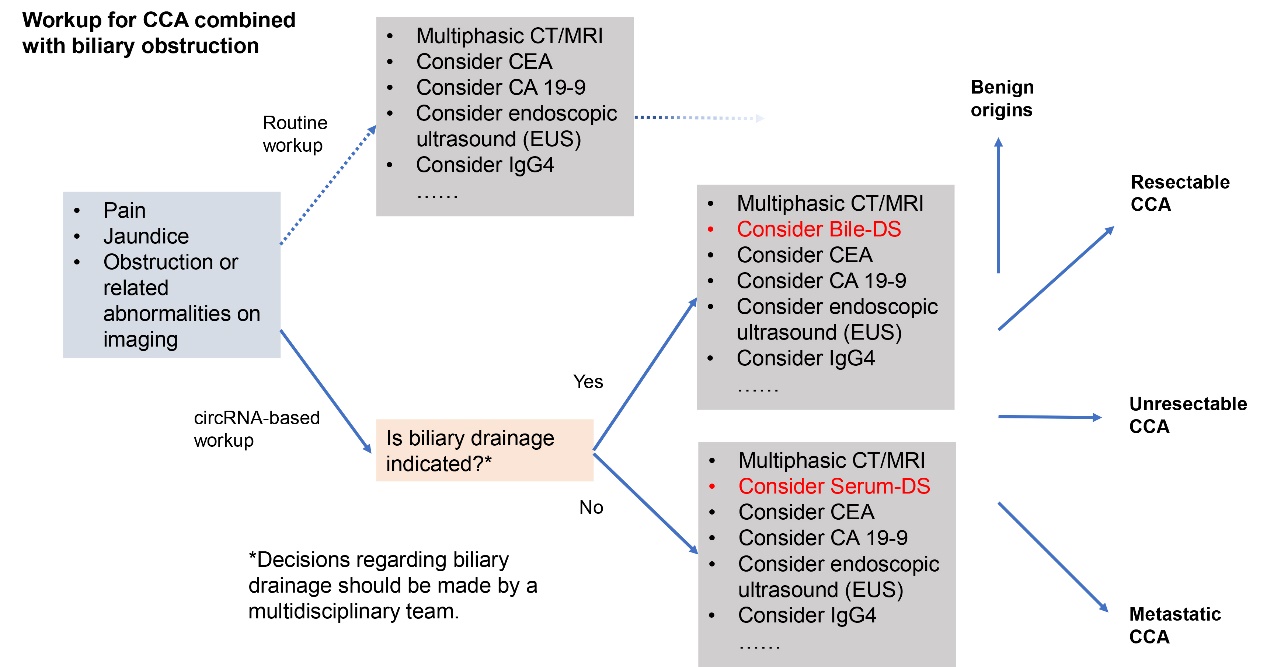


**Supplementary Fig. 20 Schematic workflow of bile or serum-based detection strategy for biliary obstruction.**

**Supplementary Table. 1** Postoperative outcome between high/low Bile-DS groups and high/low Serum-DS groups.

| Variables | Training cohort grouped by Bile-DS/Serum-DS levels | | | | | | |
| --- | --- | --- | --- | --- | --- | --- | --- |
|  | Low Bile-DS (n=26) | High Bile-DS (n=25) | pValue |  | Low Serum-DS (n=42) | High Serum-DS (n=41) | pValue |
| Histologic grade |  |  | 0.009 |  |  |  | 0.036 |
| G1 | 5 (19.2%) | 0 (0.0%) |  |  | 5 (11.9%) | 2 (4.9%) |  |
| G2 | 17 (65.4%) | 13 (52.0%) |  |  | 30 (71.4%) | 22 (53.7%) |  |
| G3 | 4 (15.4%) | 12 (48.0%) |  |  | 7 (16.7%) | 17 (41.5%) |  |
| CCA subtypes |  |  | 0.096 |  |  |  | 0.538 |
| iCCA involving the hepatic hilus | 3 (11.5%) | 9 (36.0%) |  |  | 8 (19.0%) | 10 (24.4%) |  |
| pCCA | 14 (53.8%) | 8 (32.0%) |  |  | 18 (42.9%) | 20 (48.8%) |  |
| dCCA | 9 (34.6%) | 8 (32.0%) |  |  | 16 (38.1%) | 11 (26.8%) |  |
| Tumor diameter |  |  | 1.000 |  |  |  | 0.719 |
| <3.5cm | 11 (42.3%) | 10 (40.0%) |  |  | 18 (42.9%) | 15 (36.6%) |  |
| >3.5cm | 15 (57.7%) | 15 (60.0%) |  |  | 24 (57.1%) | 26 (63.4%) |  |
| Tumor margin |  |  | 0.026 |  |  |  | 0.108 |
| R1 | 0 (0.0%) | 6 (24.0%) |  |  | 3 (7.1%) | 9 (22.0%) |  |
| R0 | 26 (100.0%) | 19 (76.0%) |  |  | 39 (92.9%) | 32 (78.0%) |  |
| Lymph node metastasis | |  | 0.069 |  |  |  | 0.011 |
| No | 18 (69.2%) | 10 (40.0%) |  |  | 31 (73.8%) | 18 (43.9%) |  |
| Yes | 8 (30.8%) | 15 (60.0%) |  |  | 11 (26.2%) | 23 (56.1%) |  |
| Number of positive lymphnodes | |  | 0.151 |  |  |  | 0.023 |
| 0 | 19 (73.1%) | 11 (44.0%) |  |  | 32 (76.2%) | 21 (51.2%) |  |
| 1 | 4 (15.4%) | 5 (20.0%) |  |  | 6 (14.3%) | 7 (17.1%) |  |
| 2 | 3 (11.5%) | 5 (20.0%) |  |  | 3 (7.1%) | 6 (14.6%) |  |
| 3 | 0 (0.0%) | 3 (12.0%) |  |  | 0 (0.0%) | 7 (17.1%) |  |
| 4 | 0 (0.0%) | 1 (4.0%) |  |  | 1 (2.4%) | 0 (0.0%) |  |
| AJCC TNM staging |  |  | 0.022 |  |  |  | 0.011 |
| Ⅰ | 9 (34.6%) | 1 (4.0%) |  |  | 13 (31.0%) | 5 (12.2%) |  |
| Ⅱ | 6 (23.1%) | 8 (32.0%) |  |  | 14 (33.3%) | 8 (19.5%) |  |
| Ⅲ | 11 (42.3%) | 16 (64.0%) |  |  | 15 (35.7%) | 28 (68.3%) |  |
| Portal vein invasion |  |  | 0.326 |  |  |  | 0.433 |
| Yes | 7 (26.9%) | 11 (44.0%) |  |  | 11 (26.2%) | 15 (36.6%) |  |
| No | 19 (73.1%) | 14 (56.0%) |  |  | 31 (73.8%) | 26 (63.4%) |  |
| Macrovascular invasion | |  | 0.921 |  |  |  | 1.000 |
| Yes | 8 (30.8%) | 9 (36.0%) |  |  | 13 (31.0%) | 13 (31.7%) |  |
| No | 18 (69.2%) | 16 (64.0%) |  |  | 29 (69.0%) | 28 (68.3%) |  |
| Microvascular invasion | |  |  |  |  |  | 1.000 |
| Yes | 5 (19.2%) | 8 (32.0%) | 0.469 |  | 10 (23.8%) | 9 (22.0%) |  |
| No | 21 (80.8%) | 17 (68.0%) |  |  | 32 (76.2%) | 32 (78.0%) |  |

Pearson's chi-squared test was applied for categorical variables, presented as number (percentage).

**Supplementary Table. 2** Baseline characteristics of patients in the training cohort and validation cohort to establish a prognostic model.

| Covariates | Training cohort  (n=83) |  | Validation cohort  (n=42) | | | pValue | |  |
| --- | --- | --- | --- | --- | --- | --- | --- | --- |
| Sex |  | | |  |  | | 1.000 | |
| Male | 56 (67.5%) | | |  | 29 (69.0%) | |  | |
| Female | 27 (32.5%) | | |  | 13 (31.0%) | |  | |
| Age | 60 (49, 63) | | |  | 62 (52, 65) | | 0.210 | |
| CCA subtypes |  | | |  |  | | 0.621 | |
| iCCA involving the hepatic hilus | 18 (21.7%) | | |  | 7 (16.7%) | |  | |
| pCCA | 38 (45.8%) | | |  | 23 (54.8%) | |  | |
| dCCA | 27 (23.5%) | | |  | 12 (28.6%) | |  | |
| CA19-9 | 84.3 [21.3, 524.0] | | |  | 283.0 [66.1, 767.0] | | 0.017 | |
| CEA | 3.4 [1.9, 7.0] | | |  | 2.7 [1.8, 4.7] | | 0.172 | |
| CA125 | 18.7 [10.5, 35.2] | | |  | 21.8 [13.1, 68.6] | | 0.271 | |
| AFP | 4.0 [2.4, 7.6] | | |  | 2.7 [1.6, 3.5] | | 0.002 | |
| TB | 125.4 [42.7, 245.9] | | |  | 180.3 [103.2, 303.9] | | 0.081 | |
| DB | 103.8 [31.4, 216.5] | | |  | 155.5 [86.3, 246.9] | | 0.090 | |
| DB/TB | 0.82 [0.71, 0.89] | | |  | 0.81 [0.77, 0.86] | | 0.929 | |
| ALT | 69 [47, 127] | | |  | 85 [55, 105] | | 0.551 | |
| AST | 68 [42, 101] | | |  | 69 [54, 114] | | 0.441 | |
| ALB | 39 (4.3) | | |  | 36 (4.4) | | <0.001 | |

Continuous variables with normal distribution are presented as mean value (SD) while others are presented as median [IQR]. Categorical variables are presented as frequency (percentage).

**References for Supplementary Materials**

1 Théry, C., Amigorena, S., Raposo, G. & Clayton, A. Isolation and characterization of exosomes from cell culture supernatants and biological fluids. *Current protocols in cell biology* **Chapter 3**, Unit 3.22 (2006).

2 Wu, W., Zhao, F. & Zhang, J. circAtlas 3.0: a gateway to 3 million curated vertebrate circular RNAs based on a standardized nomenclature scheme. *Nucleic Acids Res* **52**, D52-d60 (2024).

3 Tastsoglou, S. *et al.* DIANA-miRPath v4.0: expanding target-based miRNA functional analysis in cell-type and tissue contexts. *Nucleic Acids Res* **51**, W154-w159 (2023).

4 Hu, L. S. *et al.* Impact of microvascular invasion on clinical outcomes after curative-intent resection for intrahepatic cholangiocarcinoma. *J Surg Oncol* **119**, 21-29 (2019).

5 Rodríguez-Perálvarez, M. *et al.* A systematic review of microvascular invasion in hepatocellular carcinoma: diagnostic and prognostic variability. *Ann Surg Oncol* **20**, 325-339 (2013).

6 Amin, M. B. *et al.* *AJCC cancer staging manual*. Vol. 1024 (Springer, 2017).
